# Single-cell sequencing analysis reveals development and differentiation trajectory of Schwann cells manipulated by *M*. *leprae*

**DOI:** 10.1371/journal.pntd.0011477

**Published:** 2023-07-21

**Authors:** Shanshan Ma, Zihao Mi, Zhenzhen Wang, Lele Sun, Tingting Liu, Peidian Shi, Chuan Wang, Xiaotong Xue, Wenjie Chen, Zhe Wang, Yueqian Yu, Yuan Zhang, Fangfang Bao, Na Wang, Honglei Wang, Qianqian Xia, Hong Liu, Yonghu Sun, Furen Zhang

**Affiliations:** Shandong Provincial Hospital for Skin Diseases & Shandong Provincial Institute of Dermatology and Venereology, Shandong First Medical University & Shandong Academy of Medical Sciences, Jinan, Shandong, China; Yale University School of Medicine, UNITED STATES

## Abstract

**Background:**

*M*. *leprae* preferentially infects Schwann cells (SCs) in the peripheral nerves leading to nerve damage and irreversible disability. Knowledge of how *M*. *leprae* infects and interacts with host SCs is essential for understanding mechanisms of nerve damage and revealing potential new therapeutic strategies.

**Methodology/Principal findings:**

We performed a time-course single-cell sequencing analysis of SCs infected with *M*. *leprae* at different time points, further analyzed the heterogeneity of SCs, subpopulations associated with *M*. *leprae* infection, developmental trajectory of SCs and validated by Western blot or flow cytometry. Different subpopulations of SCs exhibiting distinct genetic features and functional enrichments were present. We observed two subpopulations associated with *M*. *leprae* infection, a stem cell-like cell subpopulation increased significantly at 24 h but declined by 72 h after *M*. *leprae* infection, and an adipocyte-like cell subpopulation, emerged at 72 h post-infection. The results were validated and confirmed that a stem cell-like cell subpopulation was in the early stage of differentiation and could differentiate into an adipocyte-like cell subpopulation.

**Conclusions/Significance:**

Our results present a systematic time-course analysis of SC heterogeneity after infection by *M*. *leprae* at single-cell resolution, provide valuable information to understand the critical biological processes underlying reprogramming and lipid metabolism during *M*. *leprae* infection of SCs, and increase understanding of the disease-causing mechanisms at play in leprosy patients as well as revealing potential new therapeutic strategies.

## Author summary

Leprosy remains a key public health concern for WHO, particularly affecting the poorest and most disadvantaged sectors of society in the world’s least developed countries. The exploration of the mechanism of leprosy nerve injury in order to prevent the occurrence of deformity and disability has aroused great interest and extensive attention of scientists. Although some achievements have been made, as a critical phase for the propagation of *M*. *leprae*, events occurring during the early stage of *M*. *leprae* infection of human SCs are still poorly understood. In this study, we aimed to investigate SC heterogeneity and biomarkers in different *M*. *leprae* infectious stages at a single cell resolution. We established that a stem cell-like cell subpopulation was in the early stage of differentiation and could differentiate into an adipocyte-like cell subpopulation. This research provides a novel perspective on the dynamic process of host cell differentiation and metabolism regulated by *M*. *leprae* and thus increase understanding of the mechanisms underlying the causes of disability in leprosy patients as well as identifying potential new therapeutic strategies.

## Introduction

Leprosy is a chronic infectious disease caused by *Mycobacterium leprae*, which selectively destroys the skin and peripheral nerves in susceptible individuals and can lead to disability in the late stages [1]. The most serious consequence of leprosy is peripheral nerve damage, which occurs in all clinical forms of the disease [2]. Due to the lack of a method for early diagnosis, a significant proportion of newly-diagnosed leprosy patients already have various degrees of neuropathy and even visible disability when they are diagnosed [3]. It remains unclear how *M*. *leprae* infects and interacts with the host to cause nerve damage.

Previous studies have revealed that *M*. *leprae* preferentially infects Schwann cells (SCs), which are the glial cells of the peripheral nervous system [4]. In leprosy patients, intracellular *M*. *leprae* can be observed in the SCs of peripheral nerves [2]. The interaction between SCs and *M*. *leprae* has been investigated widely but the relevant mechanisms remain unclear and findings may even be contradictory [5,6]. The reasons may be either that *M*. *leprae* cannot be cultured *in vitro* or due to the difficulty of obtaining nerve biopsy samples from leprosy patients. One remarkable study identified that *M*. *leprae* could reprogram adult SCs to a progenitor/stem cell-like stage of mesenchymal traits by downregulating SC differentiation-associated genes and upregulating genes mostly involved in mesoderm development [7]. These results provided an unexpected link between cellular reprogramming and host–pathogen interaction.

However, as a critical phase for the propagation of *M*. *leprae*, events occurring during the early stage of *M*. *leprae* infection of human SCs are still poorly understood. To date, several studies have explored the infection process at single cell resolution *in vitro*, which has increased understanding of the heterogeneity of the cell population and identified novel cell markers [8,9]. Investigation of the effect of the model pathogen *Salmonella* on macrophage responses to different intracellular states revealed a spectrum of functional host states in response to growing and non-growing bacteria, as well as regulation of the polarization of macrophages in different directions in response to intracellular bacteria [8]. The dynamics of the interaction between *Flavivirus* and host cells identified several host factors specifically related to *Flavivirus* infection, including proteins involved in the endoplasmic reticulum translocon, signal peptide processing, and membrane trafficking [9].

In this study, we aimed to investigate SC heterogeneity and biomarkers in different *M*. *leprae* infectious stages at a single cell resolution. Single cell transcriptional signatures of uninfected and infected SCs during four discrete time points were defined as uninfected, early infected, intermediate infected and late infected stages. We finally identified twelve distinct clusters revealing cell heterogeneity of SCs and characterized significantly-changed cell populations during the process of *M*. *leprae* infection. Further studies identified the transition from stem cell-like SCs to adipocyte-like SCs. Our findings present a dynamic process of SC reprogramming induced by *M*. *leprae*, and thus increase understanding of the mechanisms underlying the causes of disability in leprosy patients as well as identifying potential new therapeutic strategies.

## Methods

### Cell culture and *M*. *leprae* infection

SCs (hTERT ipn02.3 2λ ATCC CRL-3392) were obtained from ATCC (Manassas, VA, USA) and cultured using Dulbecco’s Modified Eagle’s Medium (ATCC 30–2002) with 10% FBS (Gibco/Life Technologies, Carlsbad, CA, USA) and 1% L-glutamine (Gibco), under conditions of 37°C and 5% CO_2_. SCs were infected with *M*. *leprae* at a multiplicity of infection of 40:1 and incubated at 33°C.

*M*. *leprae* (Thai-53 strain) was donated by Dr. Wang Hongsheng (Institute of Dermatology, Chinese Academy of Medical Sciences, Nanjing, China). *M*. *leprae* was inoculated in footpads of athymic mice and harvested eight months after infection according to the method described by Shepard [10–12]. Obtained *M*. *leprae* was counted using a ultrasonic bacterial dispersion counter following manufacturer’s instructions (SCIENTZ-CF, Ningbo Scientz Biotechnology Co., LTD).

The bacterial viability was determined using a Live/Dead BacLight viability kit (Molecular Probes, Eugene, OR) as previously described [13–15]. In brief, *M*. *leprae* was washed with sterile normal saline (10,000 × g for 5 min) and incubated at room temperature with a final concentration of 6 μM Syto9 and 30 μM propidium iodine (PI) for 15 min. After staining, the bacteria were suspended in normal saline solution of 10% (vol/vol) glycerol and observed by laser confocal microscope (LSM980, Zeiss).

### Immunofluorescence

SCs in 12-well plates were fixed using 4% paraformaldehyde for 1 hour followed by permeabilization with 0.5% Triton X-100 for 10 min. After blocking with 5% BSA for 1 h, cells were incubated with the primary antibodies at 37°C for 2 h and secondary antibodies at room temperature for 1 h. Cell nuclei were stained with DAPI (4’,6-diamidino-2-phenylindole) for 5 min, and stained cells were imaged using a confocal laser scanning microscope (LSM980, Zeiss, Oberkochen, Germany). *M*. *leprae* were stained with PKH67 dye according to the manufacturer’s instructions (Green Fluorescent Cell Linker Kit, Sigma-Aldrich, St Louis, MO, USA) before infection.

### Transmission electron microscopy

Cells were fixed with 4% glutaraldehyde and postfixed with 1% osmium tetroxide (OsO_4_). The samples were then dehydrated in increasing concentrations of ethanol, infiltrated using standard procedures and embedded in resin. An LKB-V ultramicrotome (LKB, Bromma, Sweden) was used to cut ultrathin sections, and a JEOL-1200EX transmission electron microscope (JEOL Ltd., Tokyo, Japan) with a MORADA-G2 camera (Olympus, Tokyo, Japan) was used to acquire images.

### Antibodies for immunofluorescence, western-blotting and flow cytometry

SOX10 antibody (ab155279; Abcam, Cambridge, MA, USA; 1:200), P75NTR antibody (Abcam, ab52987; 1:50), S100 antibody (Abcam, ab52642; 1:100), MPZ antibody (#57518; Cell Signaling Technology, Danvers, MA, USA; 1:100), and Cy5 AffiniPure Goat Anti-Rabbit IgG (H+L) (111-175-144; Jackson ImmunoResearch Laboratories, West Grove, PA, USA; 1:200) were used for cellular immunofluorescence. AKR1C2 antibody (PA5-36572; Invitrogen, Carlsbad, CA, USA; 1:500), AKR1C3 antibody (Abcam, ab84327; 1:500), SCD antibody (Cell Signaling Technology, #2794; 1:1000), SREBP1 antibody (2A4) (sc-13551; Santa Cruz Biotechnology, Santa Cruz, CA, USA; 1:500), SREBP2 antibody (Abcam, ab30682; 1:500), ASPM antibody (Invitrogen, PA5-99790; 1:500), TOP2A antibody (Cell Signaling Technology, #12286; 1:1000), β-ACTIN antibody (Cell Signaling Technology, #3700; 1:1000), α-TUBULIN antibody (Cell Signaling Technology, #2125; 1:1000), GAPDH antibody (60004-1-IG; Proteintech, Rosemont, IL, USA; 1:20000), horseradish peroxidase-labeled goat anti-mouse IgG (H+L) (Affinity Purified) (ZB-5305; ZSGB-BIO Co., Ltd., Beijing, China; 1:4000), and horseradish-peroxidase-labeled goat anti-rabbit IgG (H+L) (Affinity Purified) (ZSGB-BIO, ZB-5301; 1:4000) were used for western blotting. Alexa Fluor 488 anti-human Ki-67 antibody (350508; Biolegend, San Diego, CA, USA) was used for flow cytometry.

### Single Cell RNA sequencing preparation and sequencing

Single-cell RNA sequencing and analysis were performed as described by Mi et al. [16]. Cells with viability above 80% were washed and resuspended to prepare a suitable cell concentration of 700–1200 cells/μL for 10X Genomics Chromium system. Afterwards the cells were loaded onto the 10X Chromium Single Cell Platform (Single Cell 3′ library and Gel Bead Kit v.3) and generation of gel beads in emulsion (GEMs), barcoding, GEM-RT clean-up, complementary DNA amplification, and library construction were all performed as described in our previous publication [16]. Qubit was used for library quantification. The final library was sequenced on the Illumina Nova6000 instrument using 150-base-pair paired-end reads. Mapping to GRCh38 human genome, quality control and read counting of Ensembl genes was performed using Cell Ranger software with default parameters (version 3.0.1). The gene count of the remaining cells were aggregated using the Cell Ranger command aggr without depth normalization.

### Dimensionality reduction, clustering and visualization

Seurat (version 3.2.0) R (version 3.6.3) package was employed for downstream QC, dimensionality reduction, clustering and visualization analysis. Cells detected with higher than 200 genes and fewer than 10,000 genes and less than 10% mitochondrial reads and less than 10% red cell reads were included for further analysis. Cells predicted to be potential cell doublets were excluded using the Python Scrublet package (version v0.2). The filtered expression matrix was first normalized using Seurat’s “NormalizeData” function with default parameters. The top 2000 highly variable Genes (HVGs) were identified through the function “FindVariableFeatures”. “RunHarmony” and “RunUMAP” were used to remove the batch effect and do further dimensionality reduction, respectively. Clusters were calculated by the “FindNeighbors” and “FindClusters” function with optimal resolution (0.2) on the top 30 principal components from Harmony. Uniform Manifold Approximation and Projection (UMAP) was used to do visualized. Finally, specific markers in each cluster were identified by the “FindAllMarkers” function and genes detected in the cluster with adjusted P values < 0.05 and the top 100 of log2 (foldchange) (log2FC) were considered as marker genes for the cluster.

### TF activity inference and CytoTRACE

We inferred TF activity of each cluster or sample using the Dorothea algorithm and scored the activity using the Viper inference tool. CytoTRACE is a computational framework, which is implemented in R, to predict ordered differentiation states from scRNA-seq data. The differentiation of each cluster of cells was evaluated by CytoTRACE (https://cytotrace.stanford.edu/) in order to obtain the score table of cell differentiation probability. By default, cells are colored by their CytoTRACE value.

### Pathway enrichment analysis and gene set enrichment analysis

The g:profiler software was used to conduct functional enrichment analysis including Gene Ontology (GO): biological process (BP), Kyoto Encyclopedia of Genes and Genomes (KEGG) analysis, and Reactome (REAC) analysis. Enrichment analysis for gene set, including all expressed genes, regardless of whether they were differentially expressed in either cluster, was performed with gene set enrichment analysis (GSEA) software (https://git.bioconductor.org/packages/GSEABase) based on C2.CP:KEGG gene set collections (MSigDB v7.4, broad institute, Cambridge, MA, USA). NES, normalized enrichment score. FDR, false discovery rate.

### Pseudotime analysis and RNA velocity analyses

Single-cell trajectory analysis was conducted utilizing Monocle2 (version 2.12.0) with the DDR-Tree and default parameters. Prior to conducting Monocle analysis, we selected marker genes based on the Seurat clustering result and raw expression counts of cells that passed the filtering process. We visualized the pseudotime trajectories of cells from different subpopulations with different differentiation states. Different branches may represent different states, and the cells from different subpopulations were expressed in different colors.

Monocle3 (version 0.2.3.0), which is directly available through the Seurat Wrappers R package, was utilized specifically to calculate pseudotime inference. RNA velocity was calculated using scvelo (version 0.2.3). RNA velocity was estimated using a stochastic model performed as described by La Manno et al [17]. We extracted cell identifier, PCA/UMAP coordinate, and metadata from main Seurat data object to undergo RNA velocity analysis. The Loom file was established using Velocyto software. We then embeded the cell identifier (UMIs), UMAP coordinates, and SCs subtype to AnnData with scVelo package. Spliced/unspliced counts were measured by proportions function of scVelo from AnnData formatted data. We estimated the velocity and visualized with velocity streamline of the SC subpopulation transitions using the stochastic modeling hyperparameter [18]. Arrows were plotted on an absolute scale.

### Western blot analysis

Cells were lysed with RIPA buffer (R0020; SolarBio, Beijing, China) supplemented with protease/phosphatase inhibitors (Solarbio, P1049). Equal amounts (20 μg) of protein were separated by 10% sodium dodecyl sulfate-polyacrylamide gel electrophoresis (SDS-PAGE) and then transferred to a methanol-activated polyvinylidene difluoride (PVDF) membrane (0.45 μm) (Amersham Biosciences, Chalfont St Giles, UK). After blocking in 5% skimmed milk for 1 h at room temperature, the PVDF membrane was incubated with primary antibodies overnight at 4°C. Next day the PVDF membrane was incubated with the appropriate secondary antibody for 1 h at room temperature followed by enhanced chemiluminescence development (Immobilon Western HRP Substrate, Merck, WBKLS0500) and imaged using an Amersham Imager 600.

### Flow cytometry

SCs were detached by 0.25% trypsin-EDTA treatment. As Mi described [16], cells were fixed and permeabilized with the Transcription Factor Buffer Set (BD Biosciences, Franklin Lakes, NJ, USA), followed by antibody incubation for 30 min at 4°C. Stained cells were analyzed using the FACSAria Fusion flow cytometer (BD Biosciences). FlowJo software (BD Biosciences) was used to analyze the data obtained.

## Results

### *In vitro* modelling of SCs infected by *Mycobacterium leprae*

**Fig 1:**
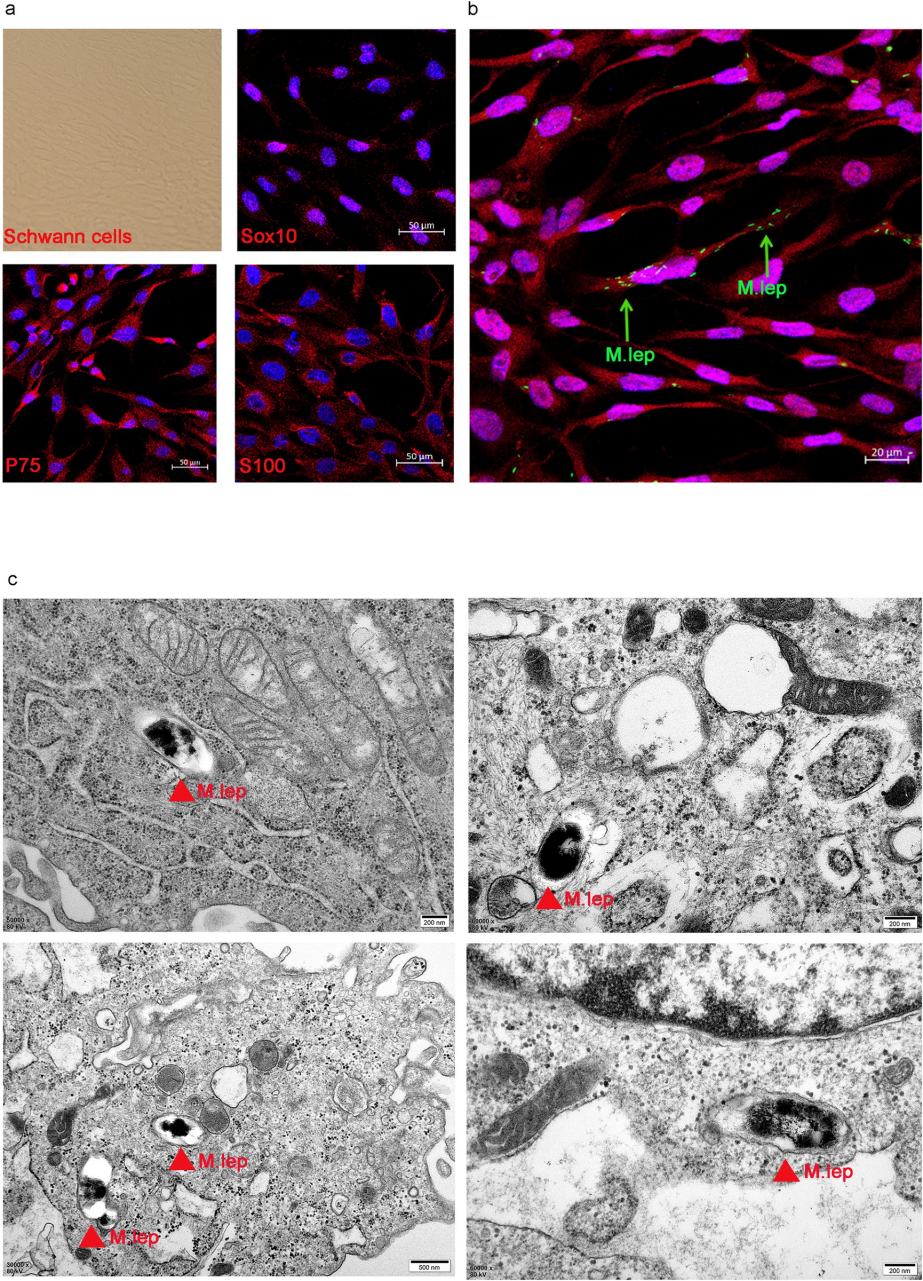
In vitro modelling of SCs infected by Mycobacterium leprae. **a.** SCs were immunolabeled with anti-Sox10 (red), anti-P75 (red), anti-S100 (red) antibodies, and counterstained with DAPI (blue) respectively. **b.** The engulfment of *M*. *leprae* by SCs was observed by confocal microscopy. *M*. *leprae* were labelled in green with the fluorescent dye PKH26. **c.** Transmission electron micrographs of SCs infected with *M*. *leprae*, with red arrow indicates engulfed *M*. *leprae*.

We employed an immortalized SC line to be infected by *M*. *leprae*. The bacterial viability was determined using a Live/Dead BacLight viability kit (S1 Fig). The cells were characterized by immunofluorescent staining with antibodies against SC-specific markers. Staining for *P75NTR*, *SOX10* and *S100* were all positive in the cell line (Fig 1A), whereas the expression of *MPZ*, which encodes an adhesion molecule necessary for normal myelination in the peripheral nervous system, was negative. These results indicated the characteristic non-myelinating nature of this SC line. The SCs were then infected by *M*. *leprae*. Engulfing of *M*. *leprae* by SCs was observed by both confocal microscopy and transmission electron microscopy (Fig 1B and 1C). All these results demonstrated the successful establishment of an infection modelling *in vitro*.

### Heterogeneity of SCs revealed by single-cell RNA sequencing

**Fig 2:**
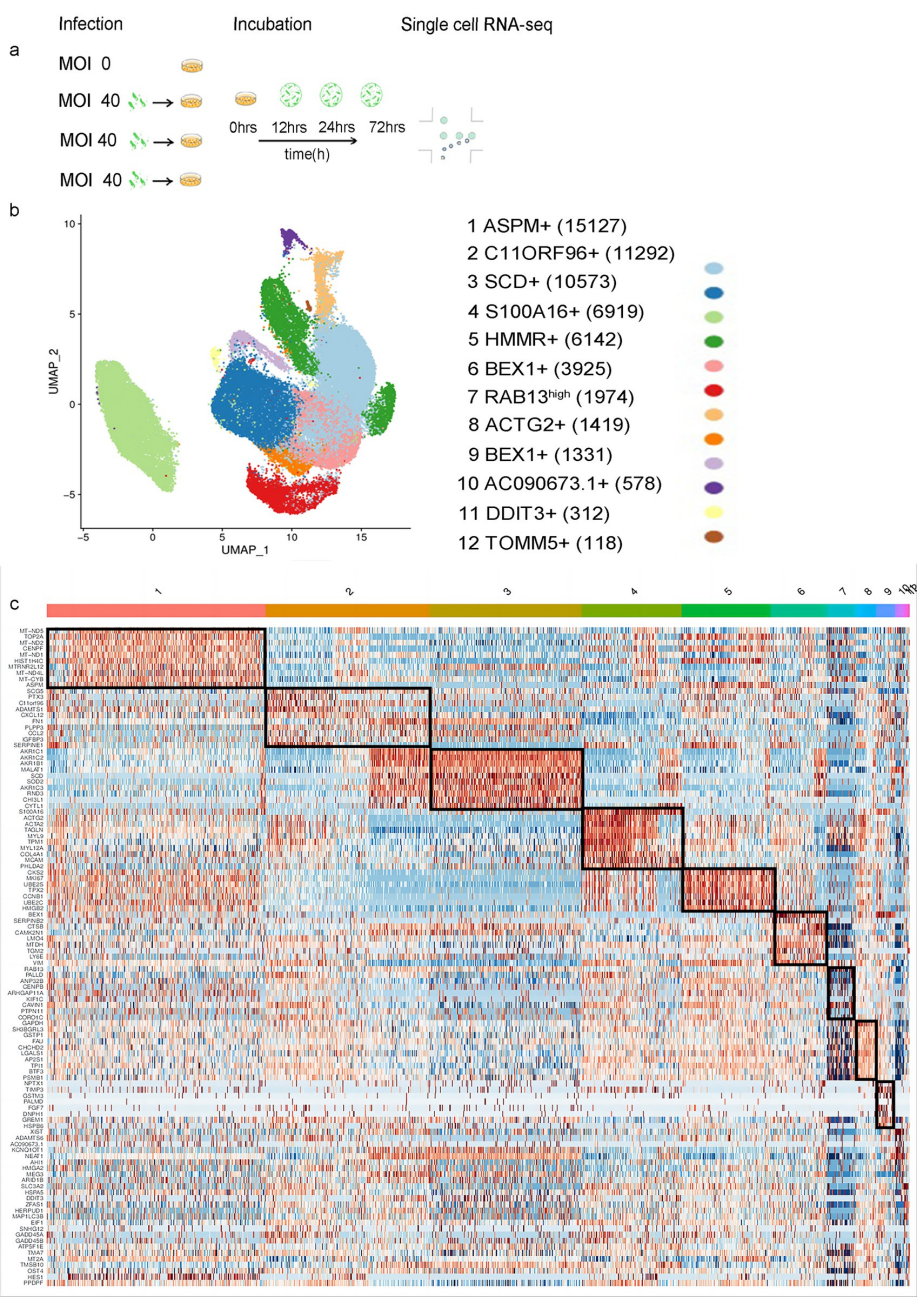
Overview of study design and single-cell RNA sequencing data. **a.** Schematic diagram for the study design and overall workflow. **b.** UMAP visualization of 12 clusters generated from 59,710 cells, discriminative marker genes for each cluster were indicated. The number of cells in each subpopulation is indicated in parentheses. **c.** Gene expression heatmap of discriminative marker genes in each subpopulation of Schwann cells.

We performed single-cell RNA-seq of all SCs, uninfected (before infection) and post-infection with *M*. *leprae*. Cells were analyzed at four time points, representing the uninfected (0 h), early (12 h), intermediate (24 h), and late (72 h) infected stages (Fig 2A). We collected one sample at each time point. After quality control, we obtained a total of 59,710 cell transcriptomes from 4 samples. Twelve transcriptionally-distinct clusters were identified (Fig 2B). The specific number of cells in each sample and each cluster was shown in S2 Fig. A standard heatmap showed the top 10 cluster-specific genes (Fig 2C). All the cluster-specific markers were provided in S1 Table.

Clusters 1 and 5 expressed high levels of stem cell marker genes and proliferative genes [19–28], such as *ASPM*, *TOP2A*, *MKI67*, *CENPF*, and *CKS2*. In addition, cluster 1 also showed high expression of mitochondrial genes, including *MT-ND5*, *MT-ND2*, *MT-ND1*, *MT-ND4L*, and *MT-CYB*, whose functions were related to the oxidative phosphorylation pathway. Cluster 2 showed high expression of *C11orf96* and several genes associated with inflammatory response and cell migration/adhesion such as *PTX3*, *CXCL8*, *FN1*, *CCL2*, *ADAMTS1*, and *CXCL18*. The gene *C11orf96* encodes a protein of unknown function but which has been reported to be upregulated after viral infection [29]. Cluster 3 showed high expression of *AKRIC1*, *AKR1C2*, *AKR1C3*, *SCD*, and *CHI3L1*, which were co-enriched to the steroid metabolism pathway by REAC pathway enrichment analysis. Cluster 4 over-expressed *S100A16*, which serves a variety of regulatory functions in numerous human pathophysiological processes, such as inflammation, adipogenesis, osteoporosis and tumor progression, and has also been considered to be a potential marker of cancer and a novel lipogenesis promoting factor, as well as being involved in neural differentiation [30–34]. Cluster 7 was found to highly express *RAB13*, a member of the Rab GTPases [35–39], playing a key role in the delivery of cargo to the plasma membrane. Clusters 4, 7, and 8 collectively expressed actin-coding genes such as *ACTA2* and *ACTG2* at a high level, which are presumably associated with actin cytoskeleton remodelling and cell migration [40–44]. Clusters 6 and 9 showed obviously high expression of *BEX1*, which is a signalling adapter molecule involved in *P75NTR* signalling, playing a role in cell cycle progression and neuronal differentiation [45]. Cluster 9 also highly expressed genes associated with inflammatory reactions and tissue remodelling, such as *CHI3L1* and *PTX3* [46,47]. The top highly expressed genes in cluster 10 were *AC090673*.*1*, *XIST*, *ADAMTS6*, and *KCNQ1OT1*. Nitrogen compound biosynthetic and metabolic process associated genes such as *ADAMTS6*, *AHI1*, *HMGA2*, and *ARID1B* were also enriched in cluster 10. Cluster 11 expressed genes related to cell death (ferroptosis/programmed cell death/apoptotic process) and autophagy such as *DDIT3*, *SlC3A2*, *MAP1lC3B*, *GADD45B*, *GADD45A*, and *SQSTM1*. Cluster 12 highly expressed *LRRC75A*, with unclear function. All the above clusters revealed the landscape of SC heterogeneity after infection by *M*. *leprae* (Fig 2C).

### Identification of SC subpopulations associated with *M*. *leprae* infection

**Fig 3:**
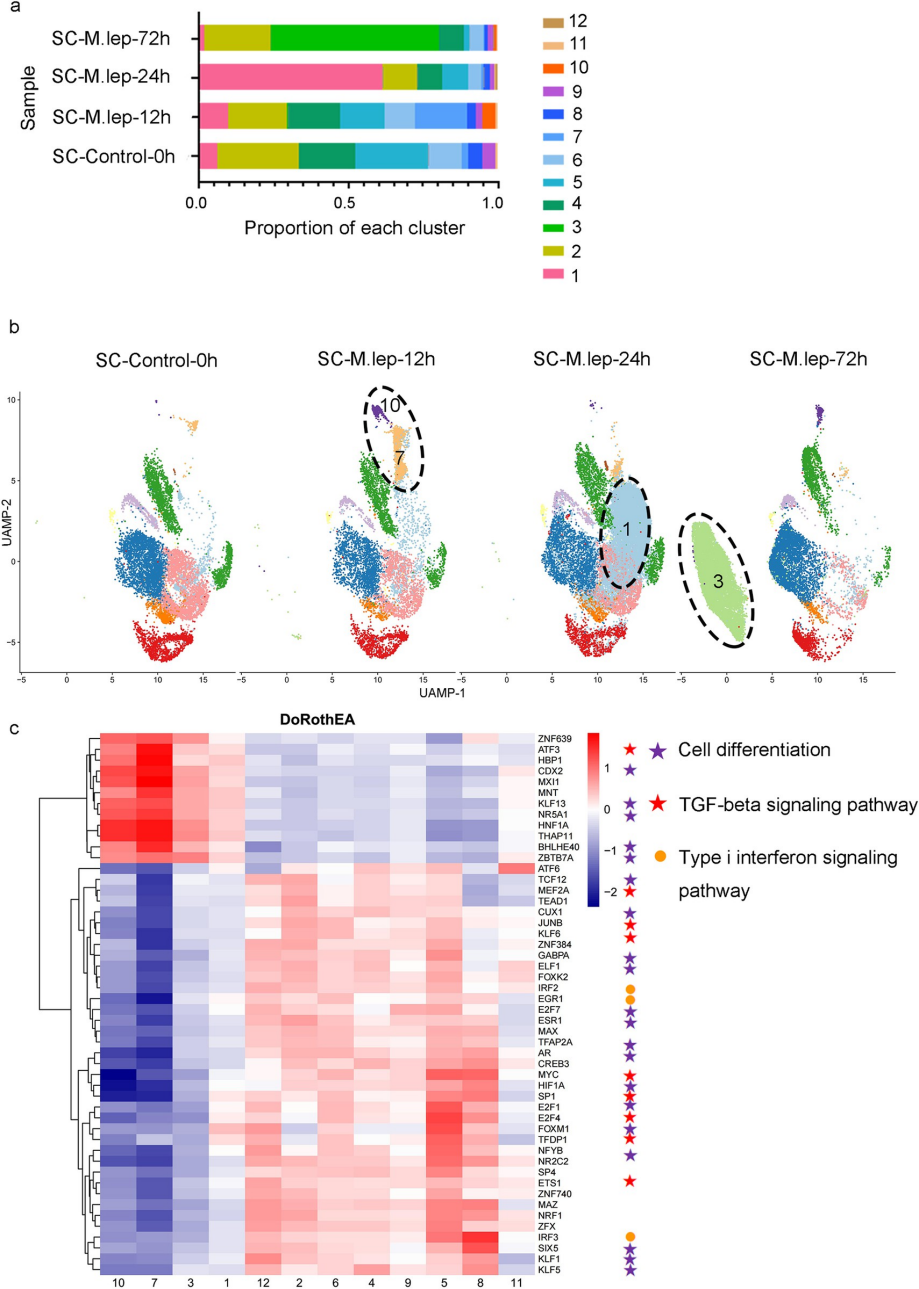
Characteristics of SC subpopulations associated with *M*. *leprae* infection. **a.** Composition for each cluster obtained in different time points of *M*. *leprae* infection. **b.** UMAP visualization of each time point of *M*. *leprae* infection. **c.** Heatmap of top 50 highly-variable TF activities among the twelve SC subpopulations; the z-scores of TF activities are color-coded.

To identify SC subpopulations specifically induced by the infection of *M*. *leprae*, we compared the cell subpopulations at different discrete time-points (Fig 3A and 3B). The proportion of cluster 1 that showed a notable increase rose from 5.9% at 0 h to 61.6% at 24 h post-infection and then decreased sharply to 1.8% at 72 h post-infection. Another remarkable finding was the presentation of cluster 3, at 72 h post-infection, which was rarely seen in other samples. Furthermore, the proportions of cluster 7 and 10 increased from 2.10% and 0.05% before infection to 17.50% and 4.10% at 12 h post-infection, respectively.

To investigate the upstream regulatory mechanisms in cell subpopulations, we inferred transcription factor (TF) activity using the Dorothea algorithm and scored the activity of each regulon using the Viper inference tool. Based on the resulting top 50 highly variant TF activity scores (Fig 3C), all subpopulations were divided into two groups: group I contained clusters whose proportion changed dramatically after *M*. *leprae* infection (clusters 1, 3, 7, 10) while other clusters were included in group II. In group I, we observed higher activity scores for *ATF3*, *CDX2*, *KLF13*, *NR5A1*, *BHLHE40*, and *ZBTB7A*, together with lower activity scores for *TCF12*, *MEF2A*, *CUX1*, *JUNB*, *ESR1*, and *MYC*. These TFs were enriched to cell differentiation and TGF-beta signalling pathway when analyzed using the protein interaction prediction database STRING. We also found decreased TF activities for *IRF2*, *EGR1*, and *IRF3* in group I, indicating a lower activation of the type I interferon signaling pathway. Similarly, TFs related to mitotic cell cycle phase transition, mitophagy and regulation of apoptotic signalling pathways got a lower activity score in group I.

In view of these results, it was suggested that four clusters in group I are more related to *M*. *leprae* infection. Cluster 1 was the main cell population in the *M*. *leprae* mid-infected stage (24 h post-infection), while during the late infected stage (72 h post-infection), the main subpopulation associated with *M*. *leprae* was cluster 3 (Fig 3A and 3B). For the early infected stage (12 h post-infection), clusters 7 and 10 were the main subpopulations associated with *M*. *leprae* infection.

### Increase of stem cell-like cell subpopulation at 24 h post-infection

**Fig 4:**
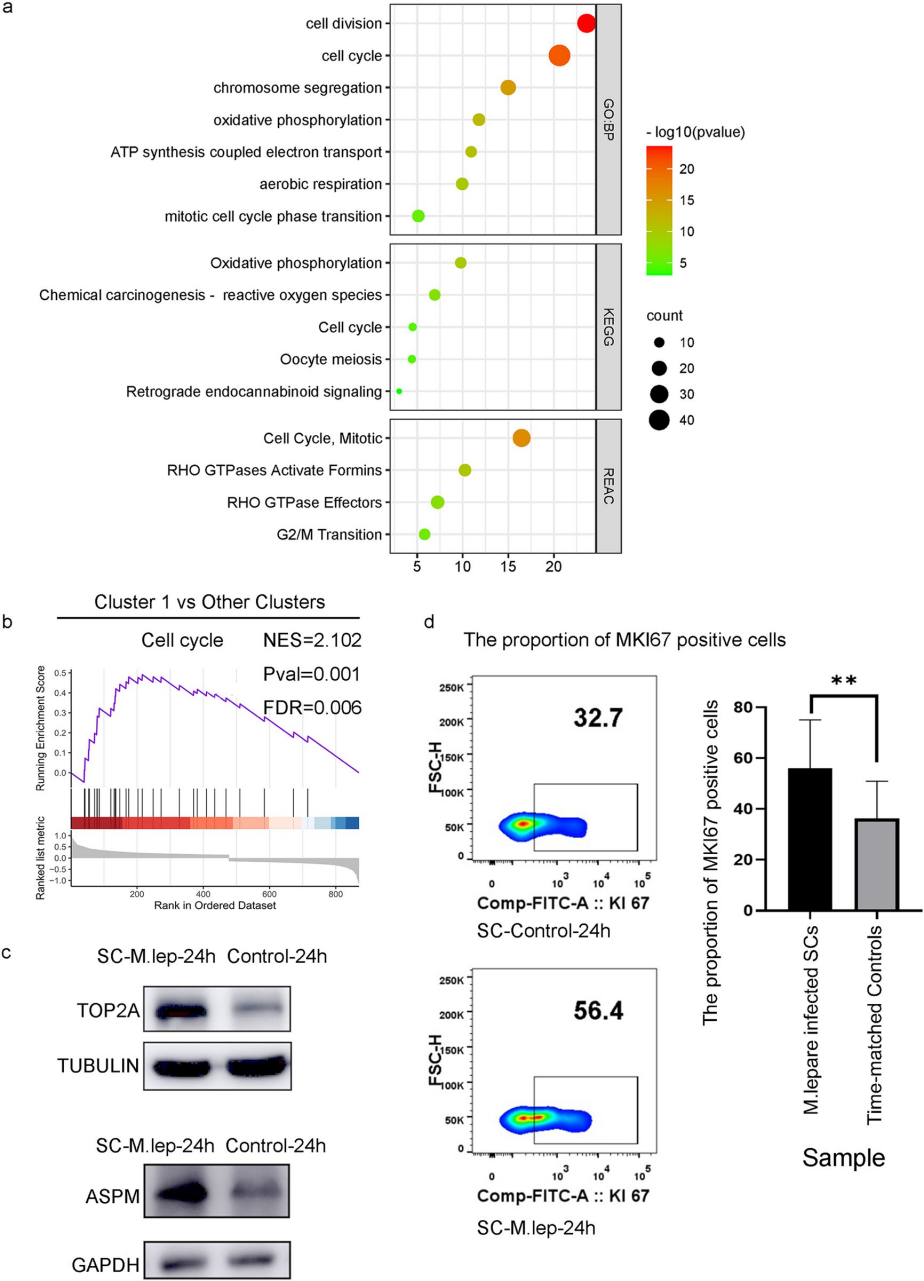
Characteristics of the stem cell-like SC subpopulation. **a.** Enrichment analysis of the top 100 highly expressed DEGs in cluster 1 compared with the other clusters. **b.** The GSEA plot of ‘cell cycle’ with higher enrichment score in cluster 1 compared with other clusters. **c.** Immunoblotting results showing the increased *ASPM* and *TOP2A* expression in SCs at 24 h post-infection compared to time-matched controls. **d.** Flow cytometry showed a significant increase of *MKI67*-positive cells among SCs at 24 h post-infection compared to time-matched controls. The significance of differences between two groups was analyzed by T-tests and non-parametric tests. Data were visualized by using Prism 8.0 GraphPad software. Statistical significance is indicated by *P* values less than 0.05. * *P* < 0.05, ** *P* < 0.01, *** *P* < 0.001, **** *P* < 0.0001.

To identify cellular characteristics at 24 h post-infection, we compared the differentially-expressed genes (DEGs) of cluster 1 to those of other clusters. Four genes (*ASPM*, *CENPF*, *MKI67*, and *TOP2A*), which are well documented as stem cell and cell proliferation markers, were overexpressed at 24 h post-infection. Therefore, cluster 1 was considered as a population of stem-like cells. In addition, pathway enrichment analysis of the top 100 highly expressed DEGs (S1 Table) using GO:BP, KEGG, and REAC databases suggested significant enrichment of cell division and cell cycle pathways (Fig 4A). Meanwhile, we evaluated cluster-specific gene set by using Gene Set Enrichment Analysis (GSEA) to convert the RNA-seq count data into biological interpretations. We investigated the functional enrichment in cluster 1 compared to other clusters. Base on the KEGG-based list, we found that ‘cell cycle’ gene set was significantly enriched, with higher enrichment score in cluster 1 compared with other clusters (Fig 4B).

To validate the single-cell sequencing results, proteins were extracted from SCs infected with *M*. *leprae* for 24 h and time-matched uninfected SCs. *ASPM* and *TOP2A* were significantly overexpressed at 24 h post-infection in SCs demonstrated by western-blotting (Fig 4C). *MKI67* was measured by flow cytometry and it was also overexpressed in SCs at 24 h post-infection (Fig 4D). Thus, these cell markers were independently validated.

### Increase in the adipocyte-like cell subpopulation at 72 h post-infection

**Fig 5:**
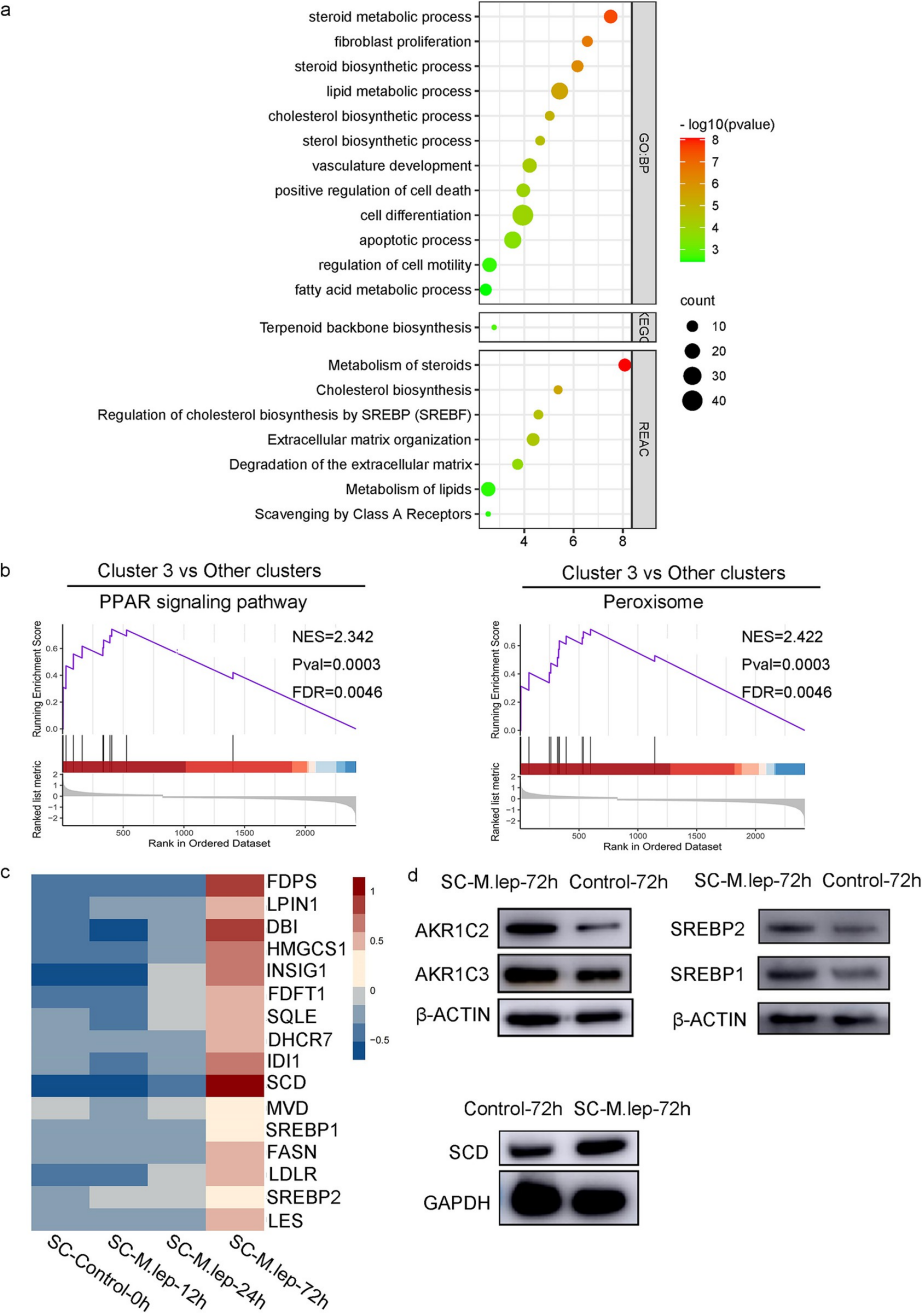
Characteristics of the adipocyte-like SC subpopulation. **a.** Enrichment analysis of the top 100 highly expressed DEGs in cluster 3 compared with the other clusters. **b.** The GSEA plot of ‘PPAR signaling pathway’ and ‘peroxisome’ with higher enrichment score in cluster 3 compared with other clusters. **c.** Heatmap of expression of genes enriched in SREBP signalling pathway. **d.** Immunoblotting results show increased expression of *AKR1C2*, *AKR1C3*, *SCD*, as well as *SREBP1* and *SREBP2* in SCs at 72 h post-infection compared to time-matched controls.

To explore the cellular characteristics at 72 h post-infection, we compared the DEGs of cluster 3 to other clusters. *SCD*, *CHI3L1* and AKR1s (*AKR1C1*, *AKR1C2*, *AKR1C3*, and *AKR1B1*) were identified as overexpressed marker genes of cluster 3. These marker genes are mainly involved in lipid metabolism, adipogenesis and tissue remodeling [48–56]. Pathway analysis of the top 100 highly expressed DEGs (S1 Table) revealed that they were enriched in lipid biosynthetic and metabolic processes, such as sterol/steroid/cholesterol/fatty acid biosynthesis and metabolism, suggesting that cluster 3 was actively involved in lipid metabolism (Fig 5A). Meanwhile, GSEA produced similar results (Fig 5B). ‘PPAR signaling pathway’ and ‘peroxisome’ gene sets were significantly enriched, with higher enrichment score in cluster 3 compared with other clusters. Genes related to the SREBP (sterol regulatory element binding proteins) signaling pathway were highly expressed at 72 h post-infection compared to other samples (Fig 5C). The overexpression of marker proteins, *AKR1C1*/*AKR1C2*, *AKR1C3*, *SCD*, *SREBP1*, and *SREBP2*, were validated by western blot (Fig 5D). Taken together, these results showed that cluster 3 could be considered as a subpopulation of adipocyte-like SCs.

### Cell state transition between cluster 1 and cluster 3

**Fig 6:**
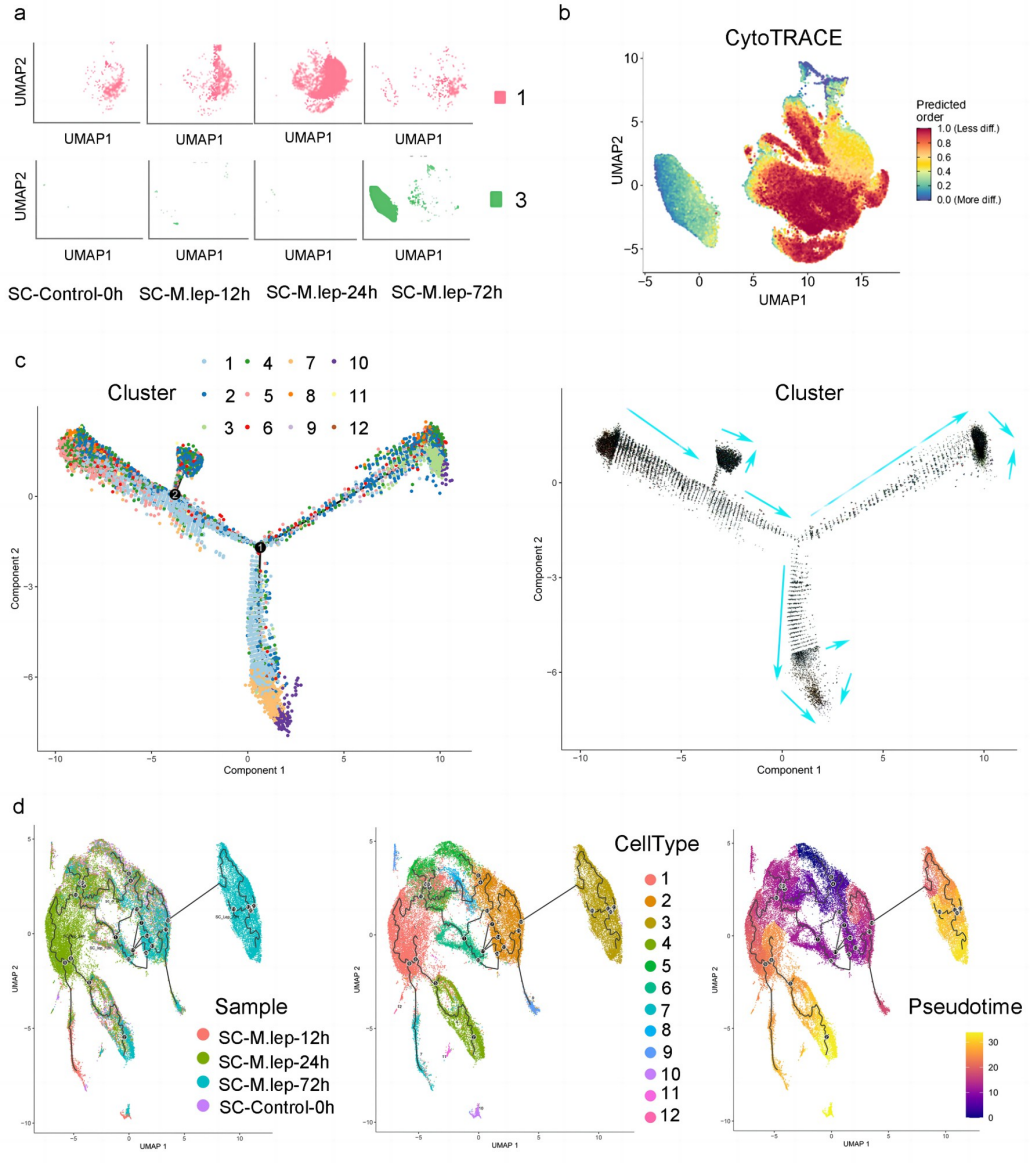
Single-cell trajectories showing the transition from cluster 1 to cluster 3. **a.** UMAP visualization of cluster 1 and cluster 3 from all the time points. **b.** The stemness of SC sub-types was analyzed by CytoTRACE and visualized by UMAP. **c.** The pseudotime analysis results of SCs, which was coloured by subpopulation. The RNA velocity analysis showing the transformation path from cluster 1 to cluster 3 (right). **d.** Pseudotime inference tree on UMAP embeddings of the samples (left) and the clusters (center) using Monocle 3; the pseudotime values are color-coded (right).

As described previously, cluster 1 decreased significantly after 72 h of infection while cluster 3 emerged (Fig 6A). To investigate the potential cell state transition between clusters, we performed CytoTRACE analysis to evaluate the differentiation states and stemness of genes in SC subpopulations. CytoTRACE is a computational framework for predicting ordered differentiation states from scRNA-seq data. The framework and UMAP distribution of CytoTRACE indicated a higher stemness of genes in cluster 1 compared to cluster 3 (Fig 6B), which suggested that the stem cell-like SCs (cluster 1) could be more likely to undergo cell state transition. Single-cell pseudotime trajectory analysis was performed to fit the optimal trajectory curve of cell development or differentiation. We found that the cells of cluster 1 were located in different branches, showing different states of differentiation, and the cells of cluster 3 were at the end of differentiation (Fig 6C, left). The RNA velocity analysis was performed to identify the directionality of cell differentiation trajectories. From the direction shown by the arrows, we found cluster 1 was in the early stage of differentiation and could differentiate into cluster 3 (Fig 6C, right).

To further decipher the transcriptional changes occurring in SCs, we conducted pseudo-temporal inference using the UMAP embedding two-dimensional space of the SC subsets (Fig 6D). The pseudotime tree revealed a continuous trajectory from uninfected to early, middle stages of infection states, and a marked transition to the late stages of infection (Fig 6D, left). Meanwhile, a pseudotime tree of clusters showed continuous tracks of other clusters, as well as a distinct transition from the other clusters to cluster 3 (Fig 6D, center). This trajectory was correlated to pseudotime values (Fig 6D, right). This result further supports the transformation of cluster 1 to cluster 3.

## Discussion

SCs serve as a reservoir for *M*. *leprae* after peripheral nervous system infection, which is central to the pathogenesis of nerve injury in leprosy [57]. Previous studies have elucidated several potential mechanisms of leprosy neuropathy, including *M*. *leprae* adhesion to SCs, endocytosis, axon atrophy, and demyelination [58]. Identification of the precise mechanism after infection remains challenging [59]. Development of single-cell sequencing technology has provided the technical potential to identify diverse cell subpopulations of SCs after infection with *M*. *leprae*. In this study, we applied single-cell RNA-seq analysis to refine the characterization of SC heterogeneity after *M*. *leprae* infection *in vitro*. We identified 12 subpopulations of SCs after *M*. *leprae* infection, each with unique transcriptional signatures.

A crucial initial step in the progression of leprosy is the binding of *M*. *leprae* to invade SC [60]. Upon binding and internalization of intracellular bacterial pathogens, a multiplicity of signals is generated inside the host cell, leading to cytoskeletal reorganization and bacterial engulfment. Previous publications have reported observation of actin accumulation around individual bacteria, and greater than 50% of the SCs contained or were associated with at least one bacterium at the earliest time-point [61]. We identified cluster 7 and cluster 10 in the early stage of *M*. *leprae* infection, which showed that SCs went through cytoskeletal rearrangement, cell migration, and phagocytosis, and might be accompanied by metabolic changes, which was consistent with previous reports [60].

At the intermediate stage of infection, cluster 1 exhibited stem-like phenotypes with distinct karyokinetic and proliferative activity, with development to the dominant cell subpopulation. These results showed that *M*. *leprae* indeed reprogramed SCs into stem cell-like cells. Upon entering the cell, *M*. *leprae* “hijacks” the proliferative machinery, reactivating quiescent SCs to enter the cell cycle again [7,62]. A consequence of this manipulation on host behaviors is that more cells become susceptible to infection, contributing to the etiology of the disease [7,62].

At the late stage of infection, we found that the dominant subpopulation, cluster 3, exhibited lipid biosynthetic and metabolic process phenotypes. As the most significant markers of cluster 3, *SCD* synthesizes unsaturated fatty acids, which appear to drive lipid droplet size [56,63]. *SCD* impairment has been reported to be associated with reduced fat stores and lipid droplet size, providing insight into regulation of membrane phospholipids [56,63]. *SCD* deficiency could induce signalling that activated the PPAR-γ pathway to partition fat toward oxidation and down-regulated *SREBP-1* expression thereby reducing lipid synthesis and storage [63]. The PPAR-γ pathway has been proven to play an influential role in the foam formation of both macrophages and SCs in leprosy [2,64]. Similarly, the SREBP pathway has been implicated in the formation of lipid droplets in macrophages [65,66]. We observed increased expression of *SREBPs* 1 and 2 in SCs exposed to *M*. *leprae in vitro*, suggesting that *SREBPs* also induce a marked effect in the process of foam formation by SCs. Clinically, highly-infected SCs in lepromatous leprosy nerves exhibit a foamy, lipid-laden appearance, but the origin and nature of these lipids have remained unclear [66]. Our data showed that *M*. *leprae* had a pronounced effect on host-cell lipid homeostasis by influencing the differentiation of SCs into cells with active lipid metabolism, explaining the possible source of the lipid increase from another uncommon perspective. *M*. *leprae* infection has previously been linked to substantial changes in metabolic pathways revealed by microarray analysis [59]. Understanding the ability of intracellular pathogens to modulate host metabolic pathways has provided insight into the nature of infection [59].

We also identified the cell state transition of SCs after *M*. *leprae* infection. Pseudo-time and RNA velocity analysis revealed the transition from stem-like cells to adipocyte-like cells. Notably, alterations in host metabolism are regulated by genes related with interferon signalling, which may have implications for the host ability to defend against pathogens. A recent single-cell RNA sequencing study reported that latently HIV-1-infected T cells exhibited a dedifferentiated phenotype, characterized by the loss of T cell-specific markers and gene regulation profiles reminiscent of hematopoietic stem cells. As reported for stem cells, latently HIV-1-infected T cells efficiently forced lentiviral superinfections into a latent state and favored glycolysis [67]. These results highlight the pivotal role of host cell development and metabolism in defending against pathogen infections.

One limitation of the study is the use of SC cell line to represent the nerve damage caused by *M*. *leprae*. Due to the difficulty of obtaining nerve biopsy samples from leprosy patients, we selected cell line for further molecular analysis. The cell line hTERT ipn02.3 2λ is a Schwann cell that was isolated from the sural nerve of a normal female and was immortalized by telomerase, which showed similar properties for most of the features to primary Schwann cells [68–72]. Future study will focus on the primary Schwann cells to validate all the results.

## Conclusions

In summary, we provided a systematic time-course analysis of SC heterogeneity after infection by *M*. *leprae* at single-cell resolution. We identified two distinct subpopulations with phenotypes of stem cell-like SCs and adipocyte-like SCs. We also found that *M*. *leprae* could regulate host cell differentiation and metabolism at different stages of infection. These results provide valuable information to understand the critical biological processes underlying reprogramming and lipid metabolism during *M*. *leprae* infection of SCs.

## Supporting information

S1 Fig Fluorescence microscopy images of all bacteria. (a) Green label represented all bacteria. (b) Red label represented dead bacteria. (c) The double fluorescence mixed image.
(TIF)

S2 Fig The cell number of each cluster and each sample. (a) The cell number of each cluster. (b) The cell number of each sample.
(TIF)

S1 Table All the cluster-specific markers. (XLS)

S1 Original western blots The results of all original western blots were recorded. (DOCX)
