# Supplementary figures and images for: Single-cell sequencing analysis reveals development and differentiation trajectory of Schwann cells manipulated by M. leprae
Source: PLoS Negl Trop Dis. 2023 Jul 21;17(7):e0011477. doi: 10.1371/journal.pntd.0011477 (PMC10361531; doi:10.1371/journal.pntd.0011477)

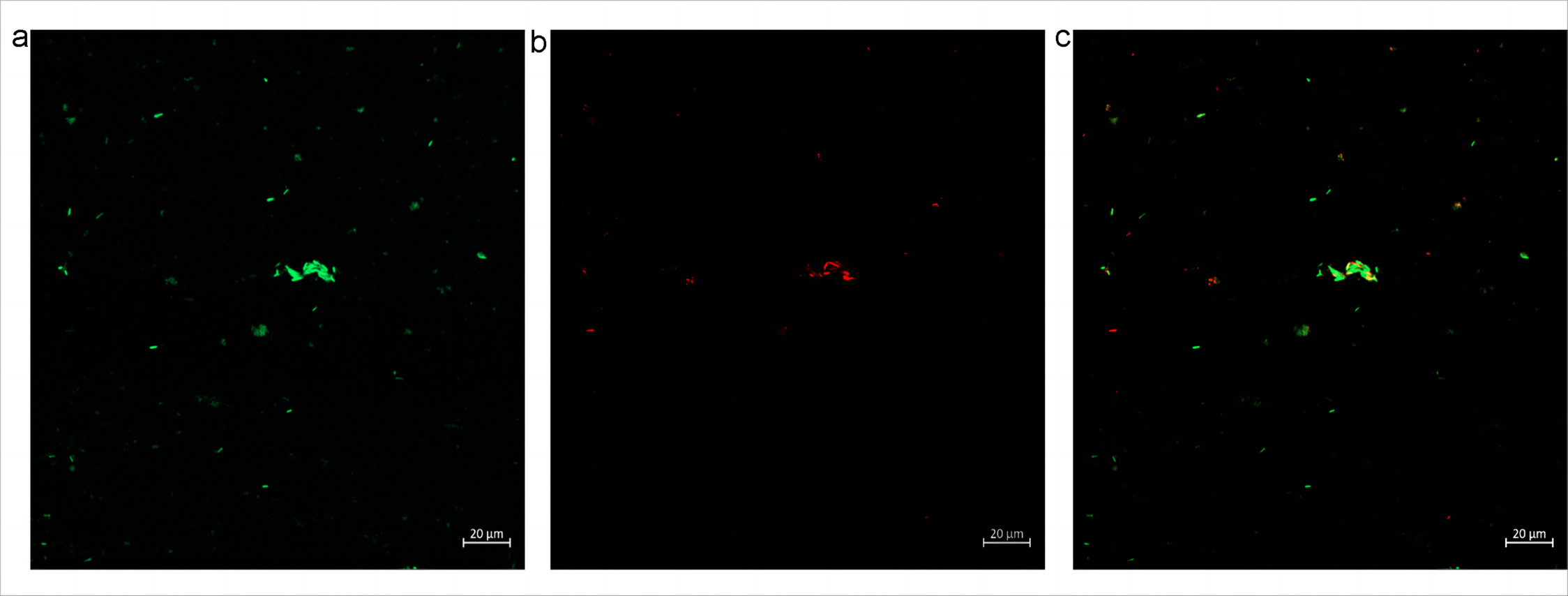

Supplement: S1 Fig — (a) Green label represented all bacteria. (b) Red label represented dead bacteria. (c) The double fluorescence mixed image. (TIF) [file pntd.0011477.s001.tif]

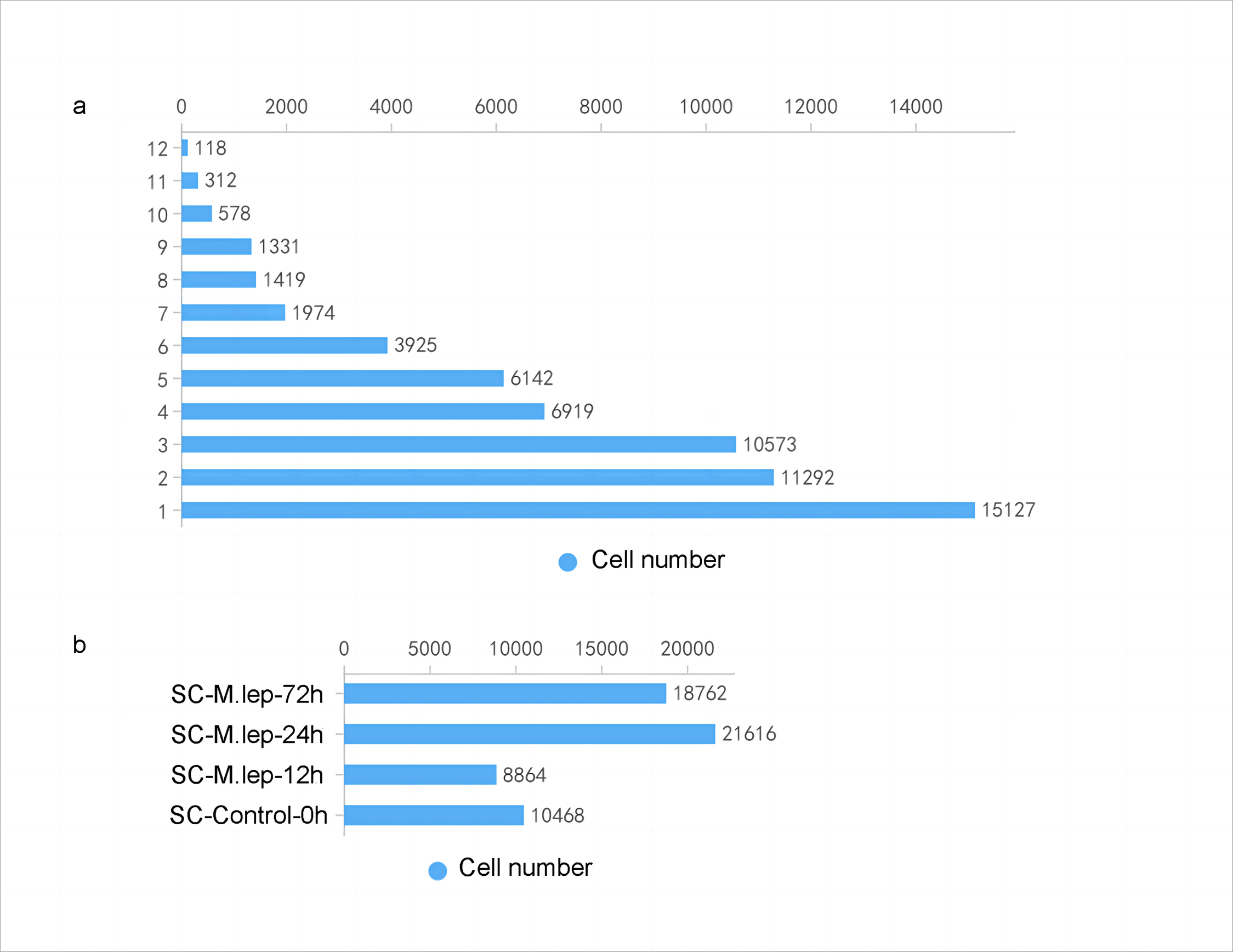

Supplement: S2 Fig — (a) The cell number of each cluster. (b) The cell number of each sample. (TIF) [file pntd.0011477.s002.tif]
