# Supplementary material for: Single-cell sequencing analysis reveals development and differentiation trajectory of Schwann cells manipulated by M. leprae
Source: PLoS Negl Trop Dis. 2023 Jul 21;17(7):e0011477. doi: 10.1371/journal.pntd.0011477 (PMC10361531; doi:10.1371/journal.pntd.0011477)
Supplement: S1 Original western blots — (DOCX) [file pntd.0011477.s004.docx]

**Original western blots**

1. TOP2A+TUBULIN

TOP2A

SC-M.lep-24h Control-24h


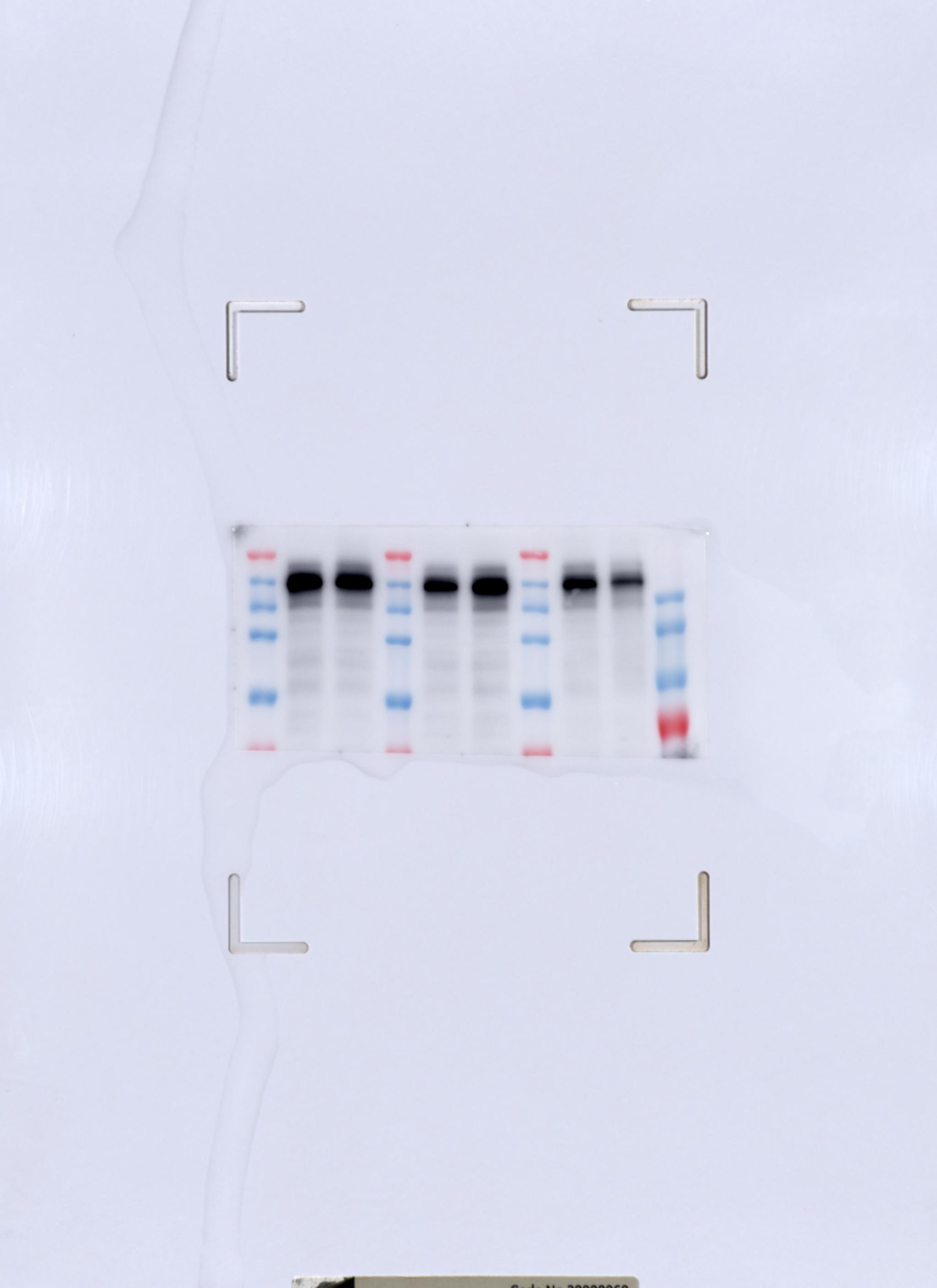


180kd

315kd

250kd

180kd

TUBULIN


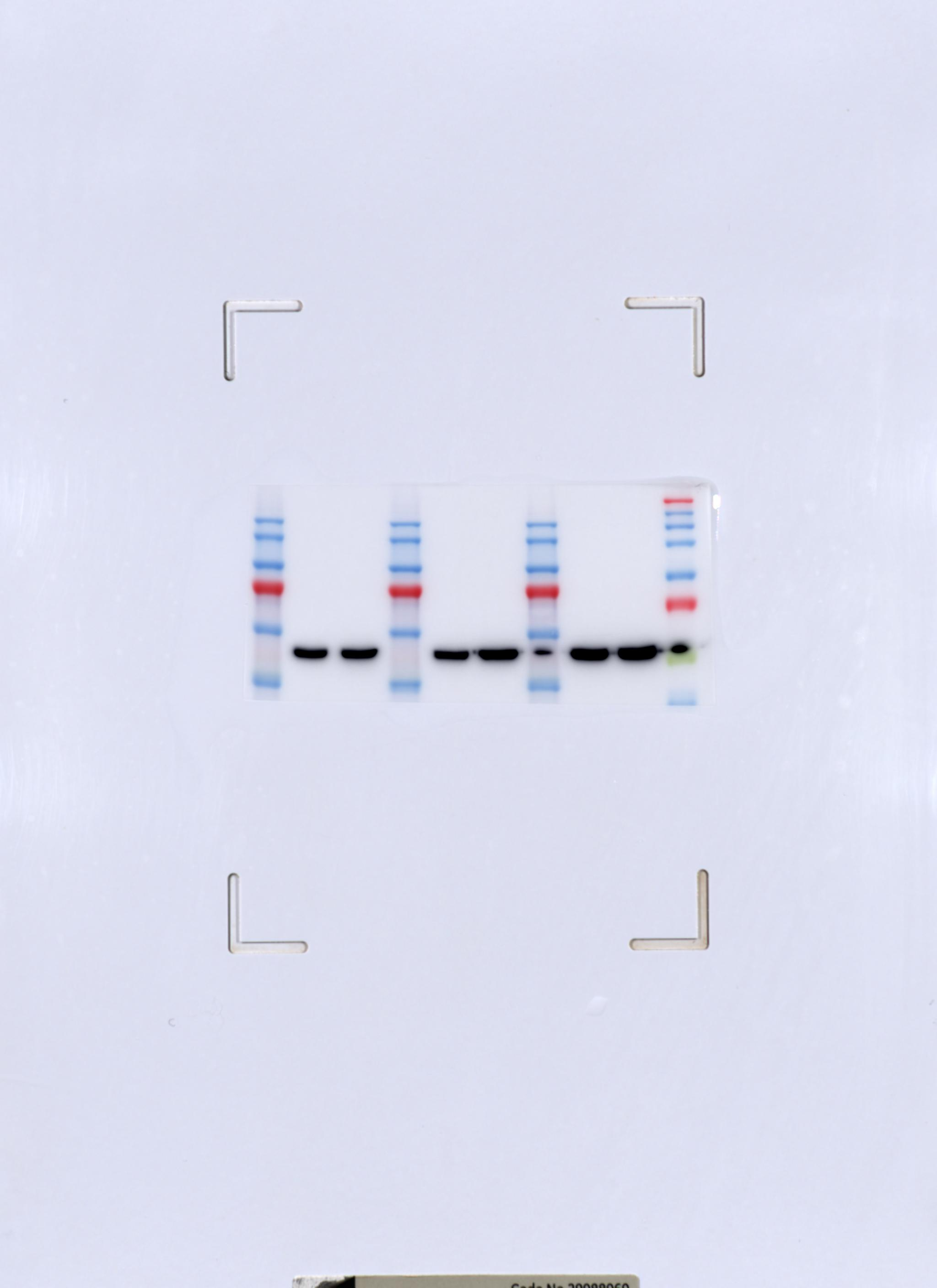


SC-M.lep-24h Control-24h

52kd

1. ASPM+GAPDH

ASPM


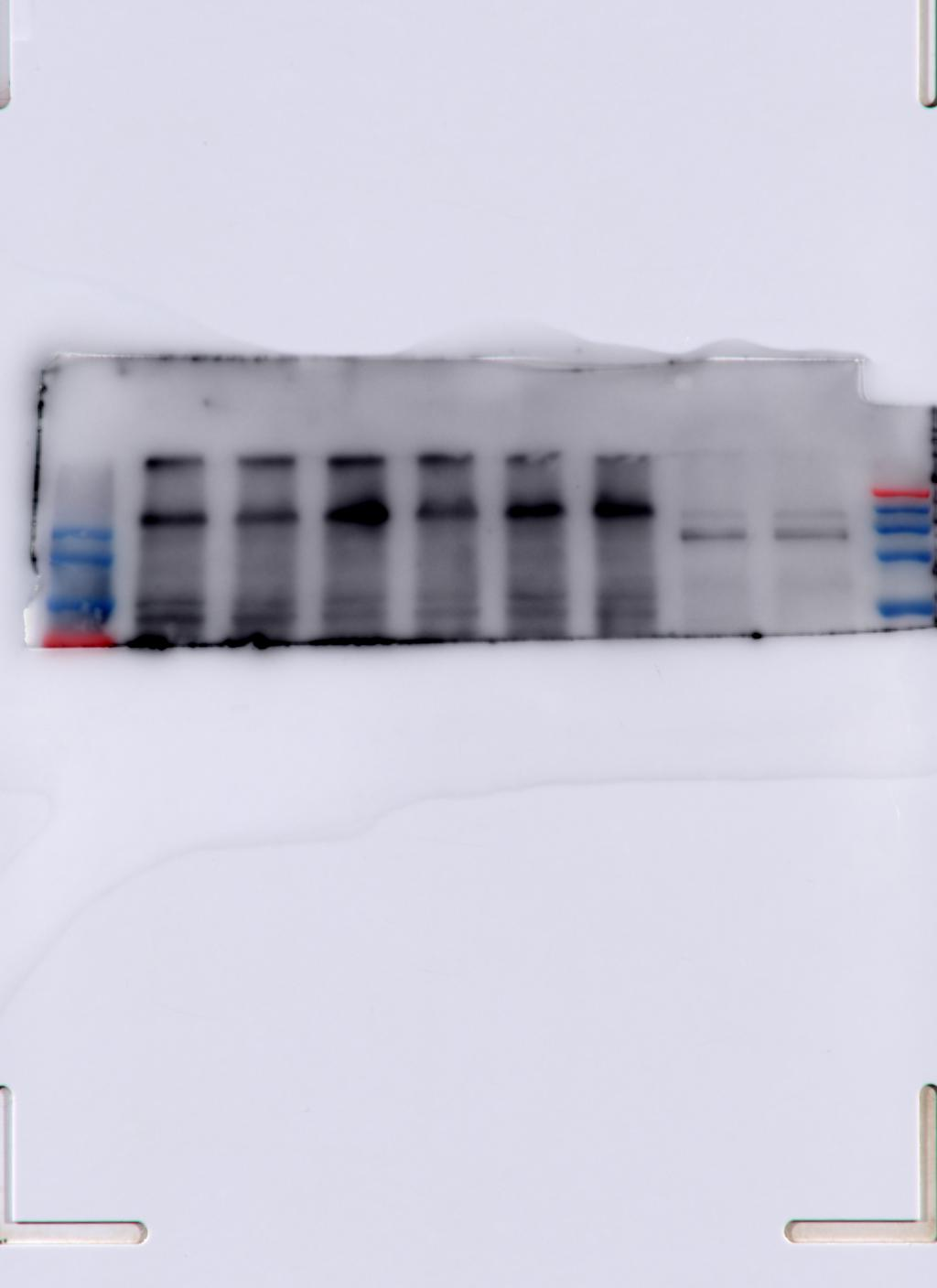


SC-M.lep-24h Control-24h

180kd

180kd

250kd

GADPH


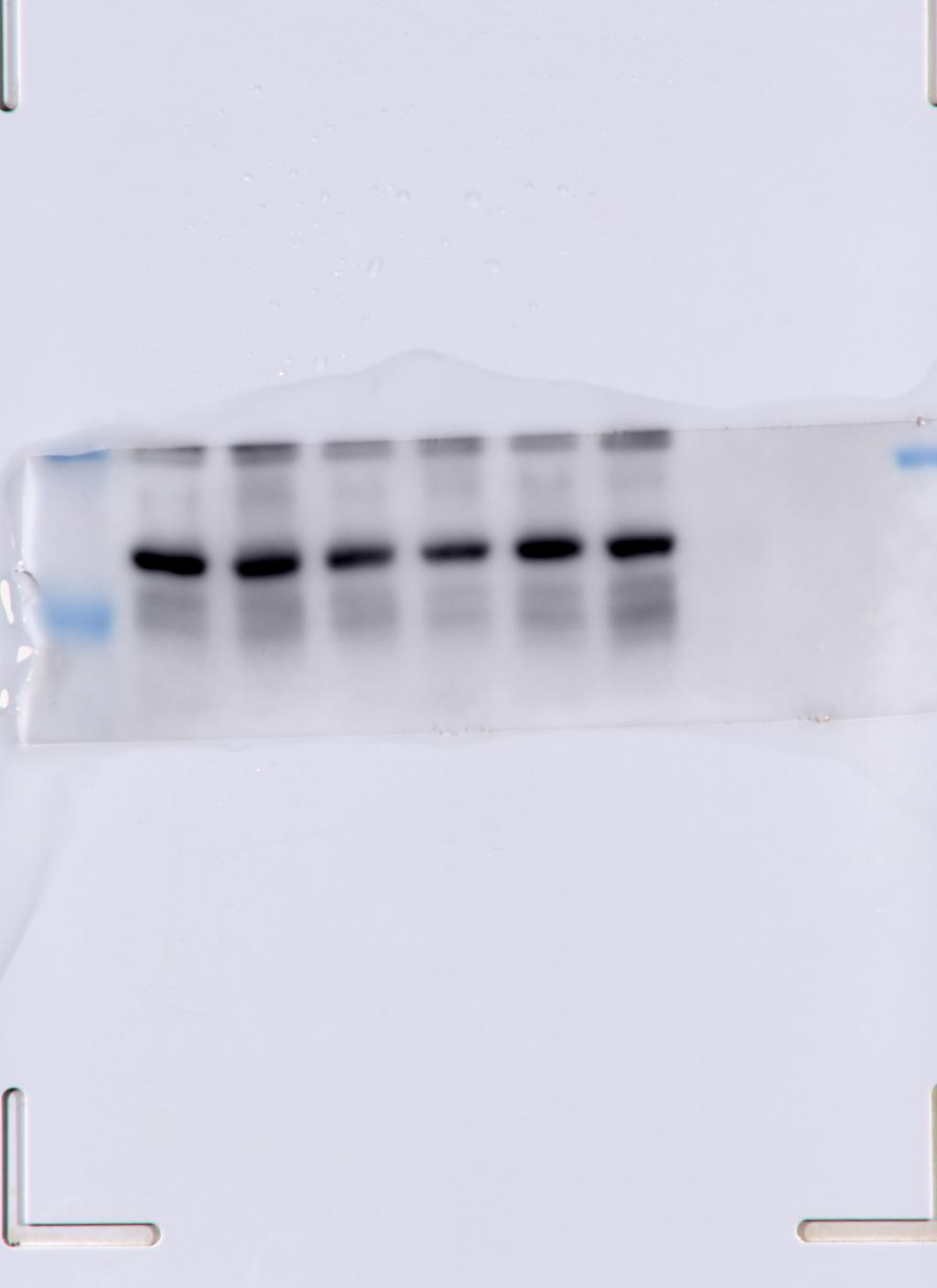


SC-M.lep-24h Control-24h

35kd

45kd

1. AKR1C2+AKR1C3+β-ACTIN

AKR1C2


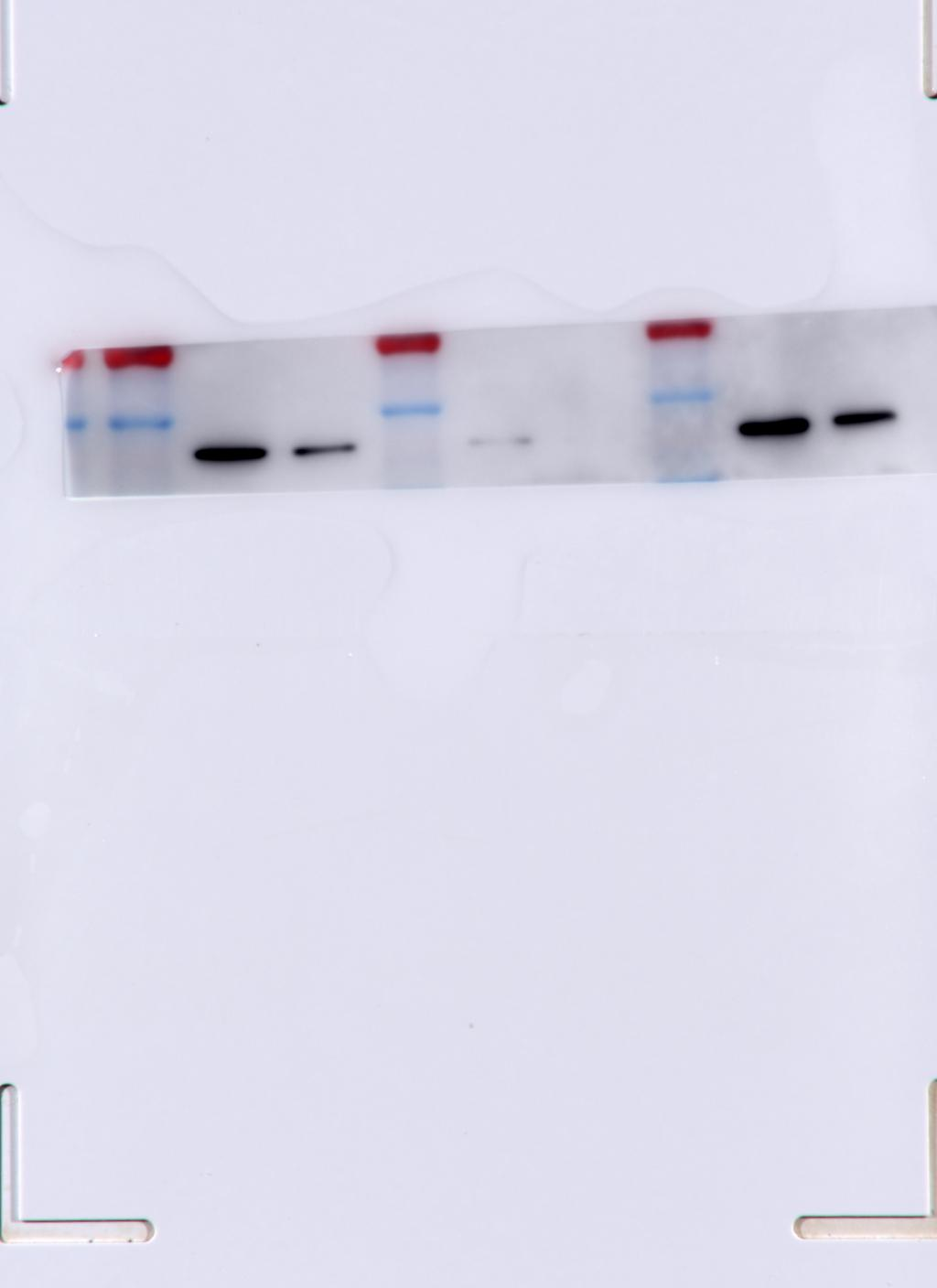


60kd

SC-M.lep-72h Control-72h

AKR1C3


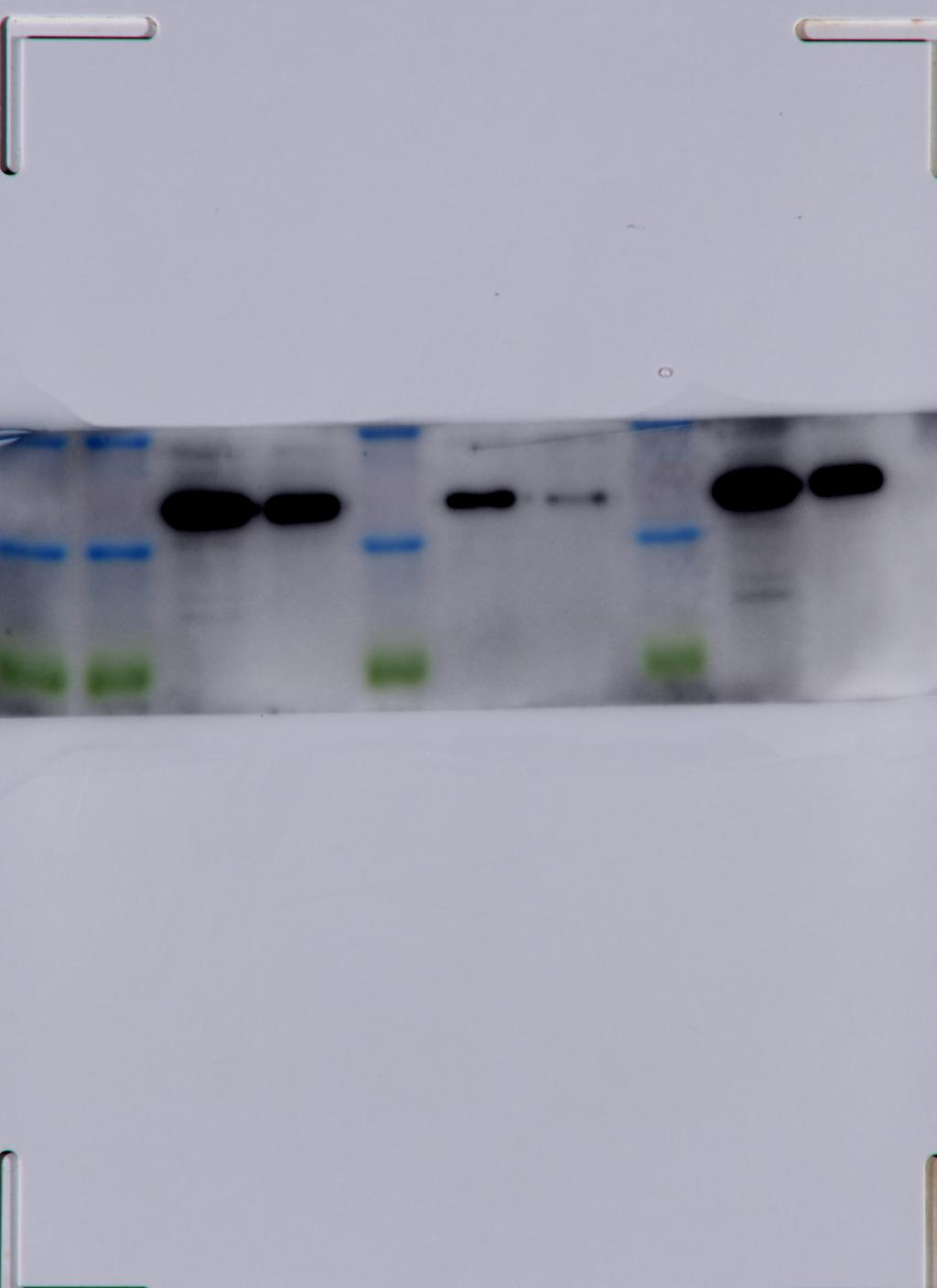


SC-M.lep-72h Control-72h

45kd

35kd

β-ACTIN


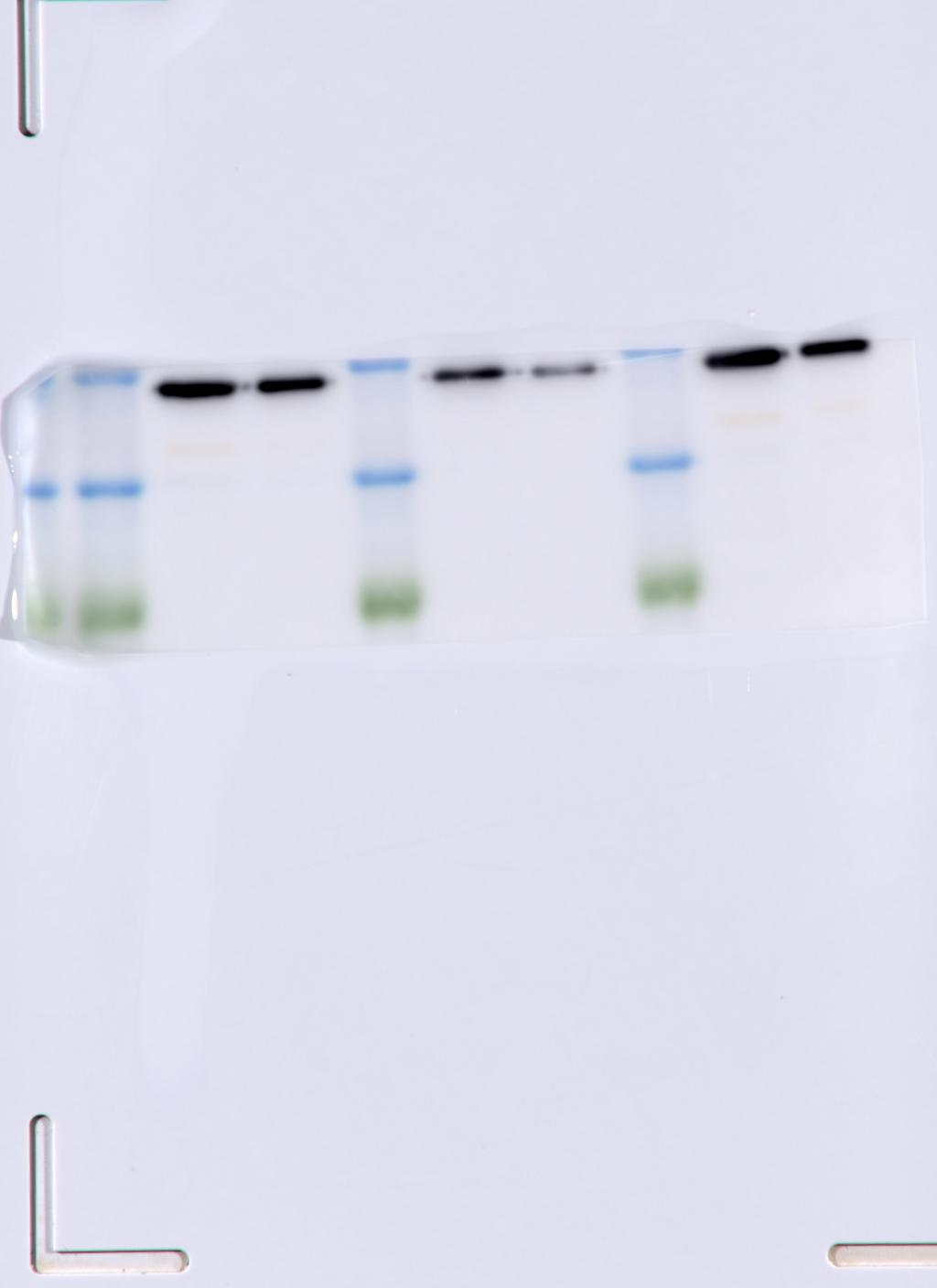


35kd

45kd

SC-M.lep-72h Control-72h

1. SREBP1+SREBP2+β-ACTIN

SREBP1


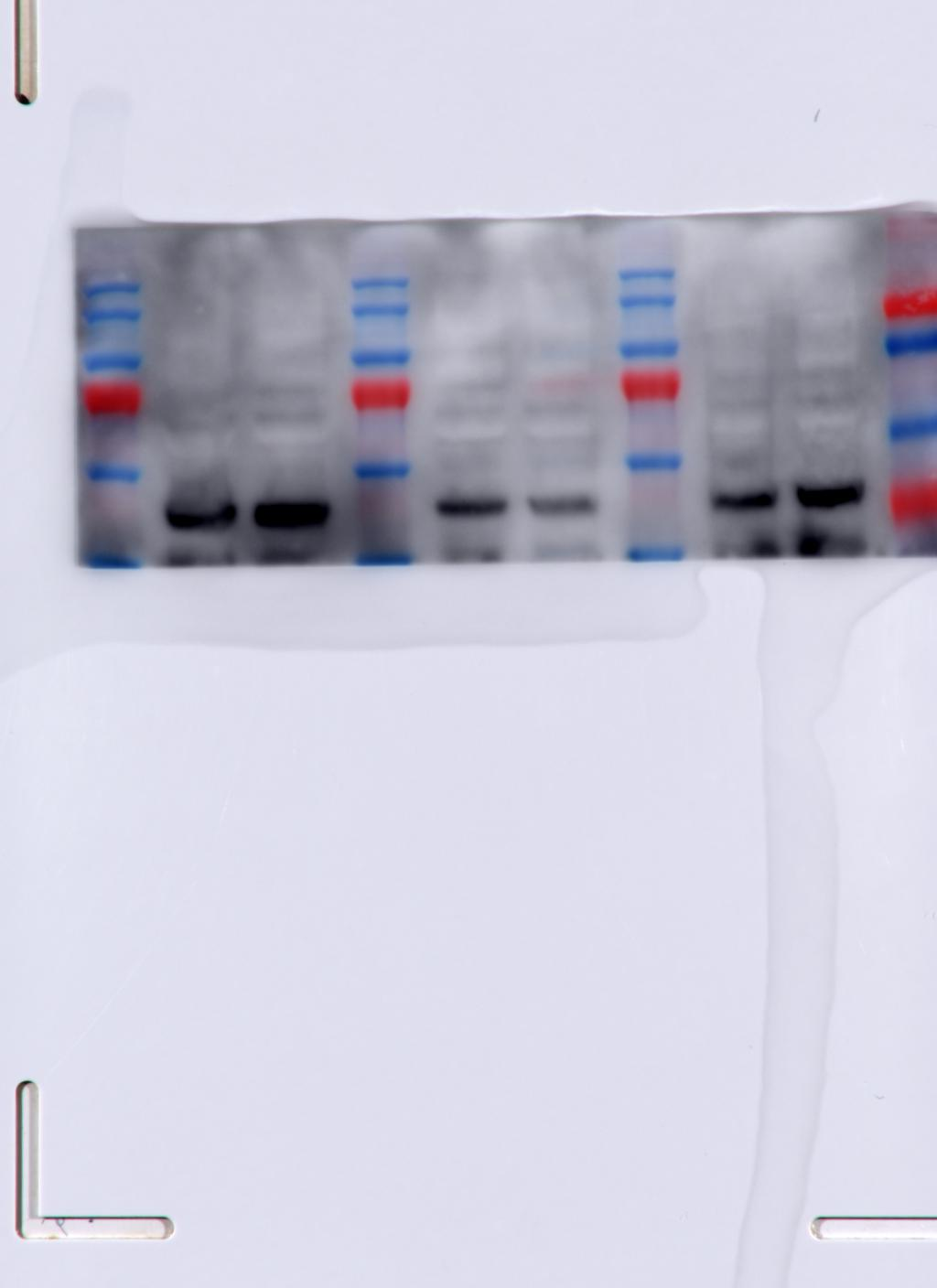


70kd

50kd

SC-M.lep-72h Control-72h

### SREBP2

###
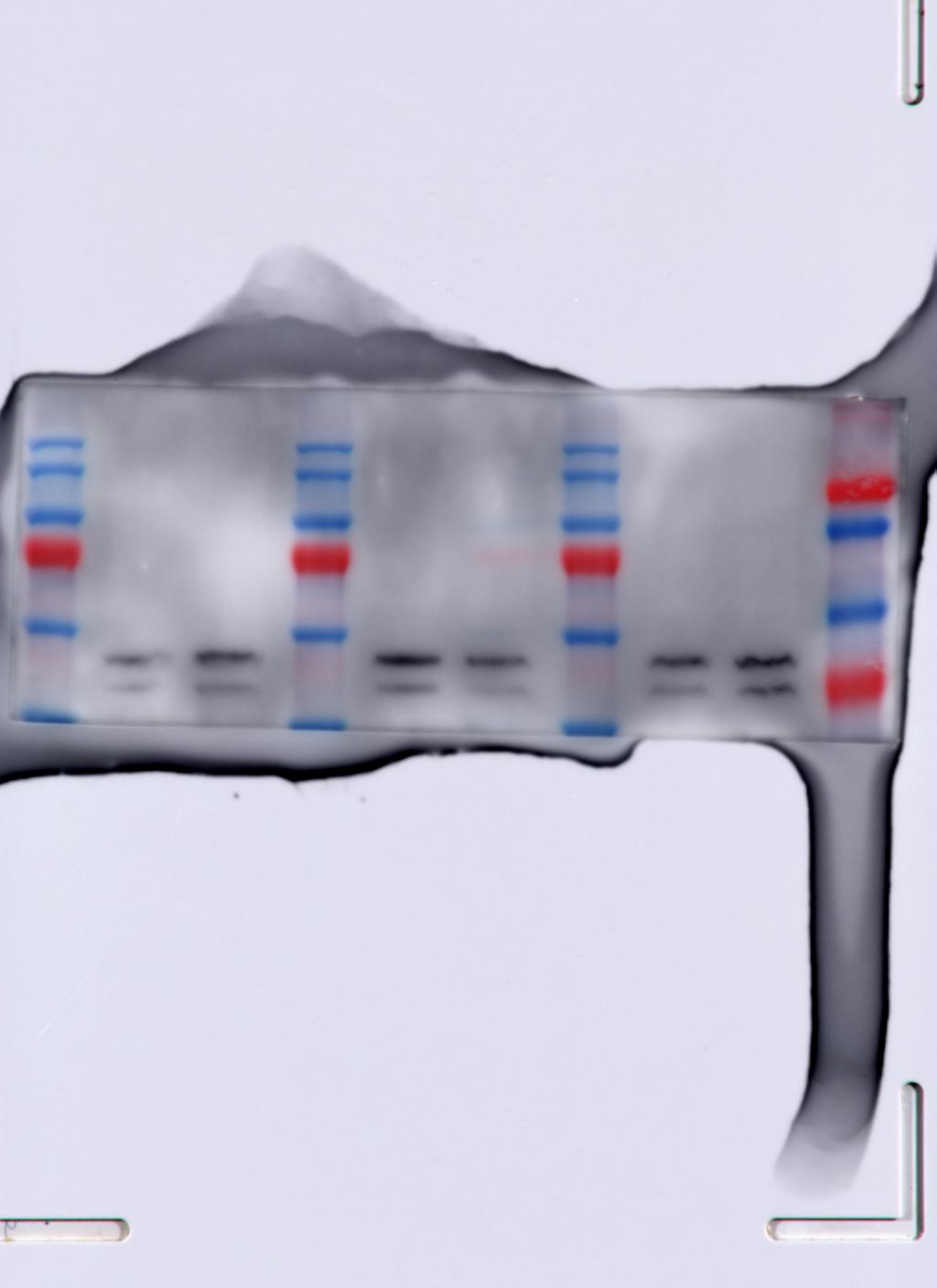


50kd

70kd

SC-M.lep-72h Control-72h

β-ACTIN


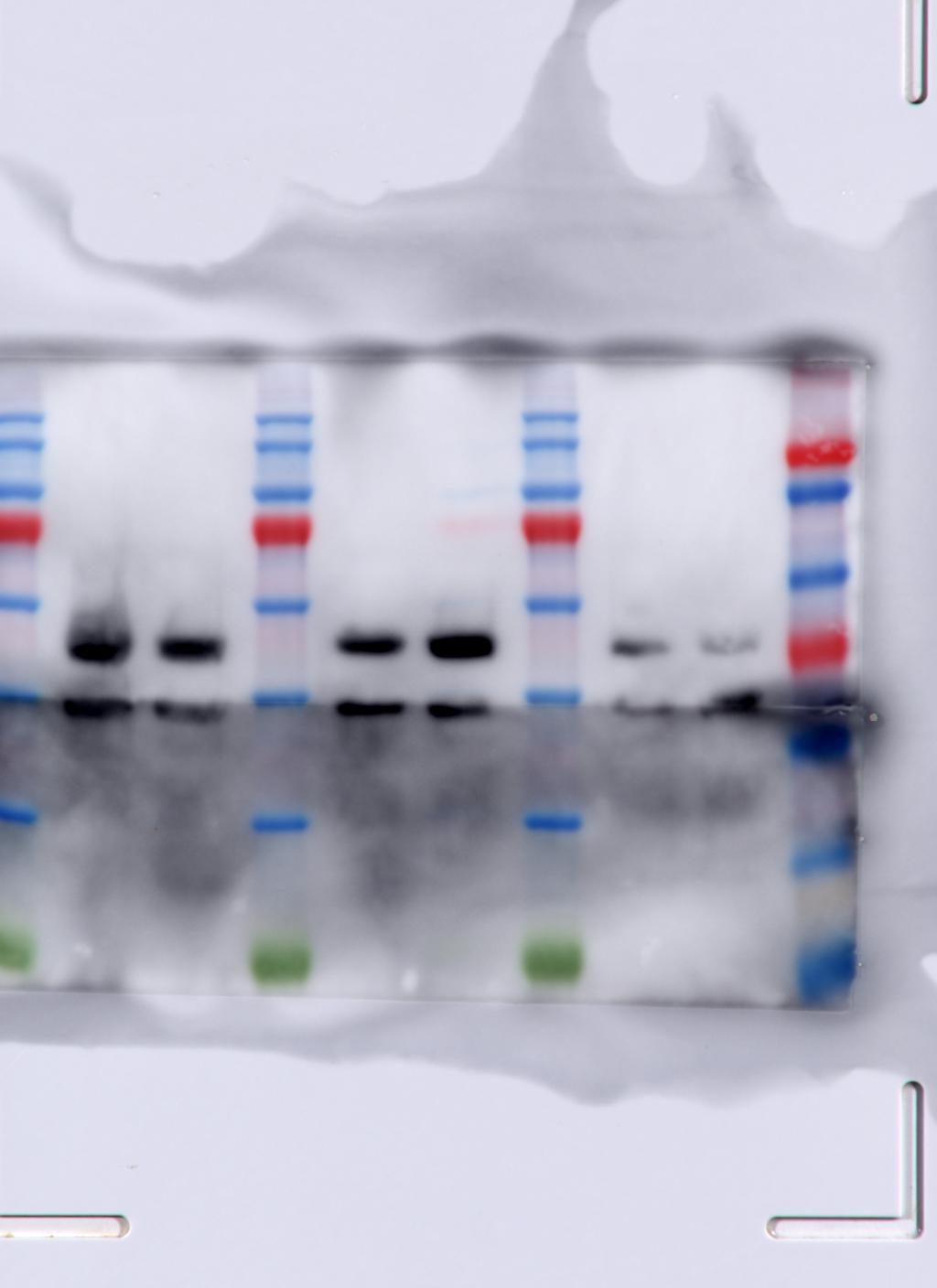


45kd

40kd

50kd

SC-M.lep-72h Control-72h

1. SCD+GAPDH

SCD

##
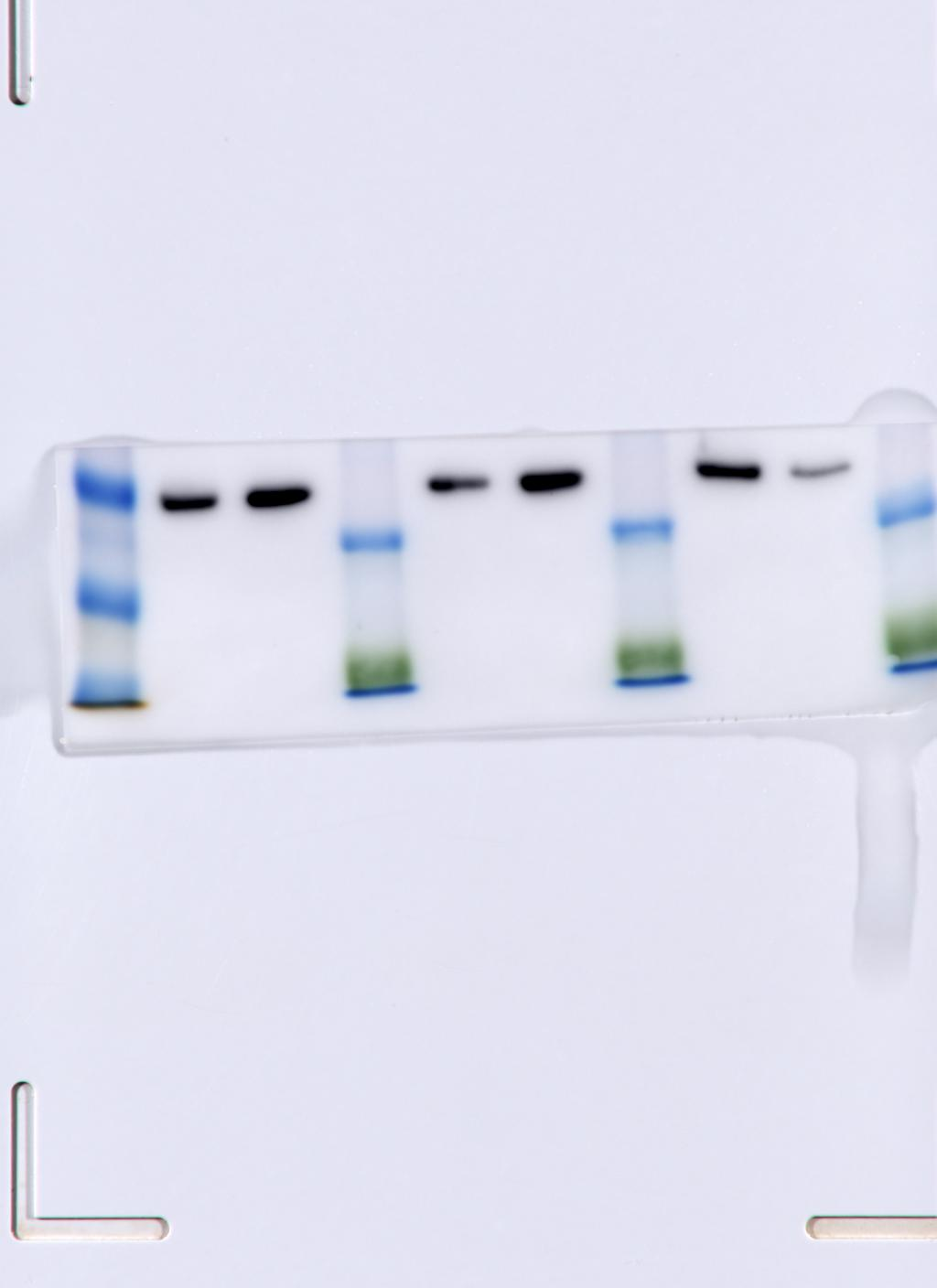


40kd

Control-72h SC-M.lep-72h

35kd

GAPDH


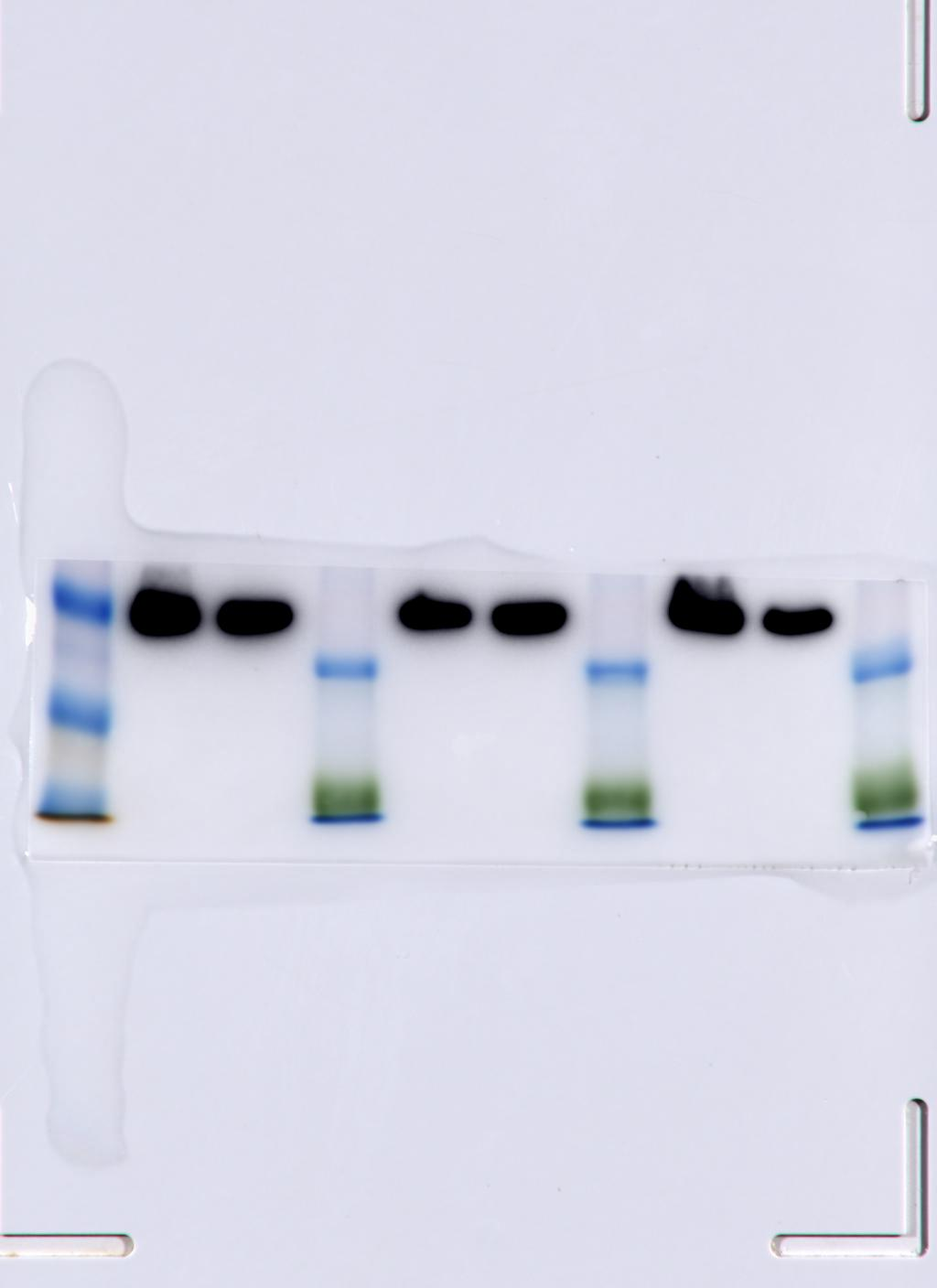


35kd

40kd

Control-72h SC-M.lep-72h
